# Molecular Bases of Catalysis and ADP-Ribose Preference of Human Mn^2+^-Dependent ADP-Ribose/CDP-Alcohol Diphosphatase and Conversion by Mutagenesis to a Preferential Cyclic ADP-Ribose Phosphohydrolase

**DOI:** 10.1371/journal.pone.0118680

**Published:** 2015-02-18

**Authors:** Alicia Cabezas, João Meireles Ribeiro, Joaquim Rui Rodrigues, Iralis López-Villamizar, Ascensión Fernández, José Canales, Rosa María Pinto, María Jesús Costas, José Carlos Cameselle

**Affiliations:** 1 Grupo de Enzimología, Departamento de Bioquímica y Biología Molecular y Genética, Facultad de Medicina, Universidad de Extremadura, Badajoz, Spain; 2 Escola Superior de Tecnologia e Gestão, Instituto Politécnico de Leiria, Leiria, Portugal; Jacobs University Bremen, GERMANY

## Abstract

Among metallo-dependent phosphatases, ADP-ribose/CDP-alcohol diphosphatases form a protein family (ADPRibase-Mn-like) mainly restricted, in eukaryotes, to vertebrates and plants, with preferential expression, at least in rodents, in immune cells. Rat and zebrafish ADPRibase-Mn, the only biochemically studied, are phosphohydrolases of ADP-ribose and, somewhat less efficiently, of CDP-alcohols and 2´,3´-cAMP. Furthermore, the rat but not the zebrafish enzyme displays a unique phosphohydrolytic activity on cyclic ADP-ribose. The molecular basis of such specificity is unknown. Human ADPRibase-Mn showed similar activities, including cyclic ADP-ribose phosphohydrolase, which seems thus common to mammalian ADPRibase-Mn. Substrate docking on a homology model of human ADPRibase-Mn suggested possible interactions of ADP-ribose with seven residues located, with one exception (Cys^253^), either within the metallo-dependent phosphatases signature (Gln^27^, Asn^110^, His^111^), or in unique structural regions of the ADPRibase-Mn family: s2s3 (Phe^37^ and Arg^43^) and h7h8 (Phe^210^), around the active site entrance. Mutants were constructed, and kinetic parameters for ADP-ribose, CDP-choline, 2´,3´-cAMP and cyclic ADP-ribose were determined. Phe^37^ was needed for ADP-ribose preference without catalytic effect, as indicated by the increased ADP-ribose *K*_m_ and unchanged *k*_cat_ of F37A-ADPRibase-Mn, while the *K*_m_ values for the other substrates were little affected. Arg^43^ was essential for catalysis as indicated by the drastic efficiency loss shown by R43A-ADPRibase-Mn. Unexpectedly, Cys^253^ was hindering for cADPR phosphohydrolase, as indicated by the specific tenfold gain of efficiency of C253A-ADPRibase-Mn with cyclic ADP-ribose. This allowed the design of a triple mutant (F37A+L196F+C253A) for which cyclic ADP-ribose was the best substrate, with a catalytic efficiency of 3.5´10^4^ M^-1^s^-1^ versus 4´10^3^ M^-1^s^-1^ of the wild type.

## Introduction

The Mn^2+^-dependent ADP-ribose/CDP-alcohol diphosphatase (ADPRibase-Mn; EC 3.6.1.53) is one of several enzymes known in mammals to hydrolyze with some degree of specificity the phosphoanhydride linkage of ADP-ribose and other nucleoside diphosphate-X (NDP-X) compounds [1, 2]. Two important peculiarities of ADPRibase-Mn, studied in the rat enzyme, are its marked preference for Mn^2+^ over Mg^2+^ as the activating cation, with a requirement of low micromolar Mn^2+^ for activity [1, 2], which is near the physiological level of the metal in animal cells [3], and its minor but unique activity on the phosphoanhydride linkage of cyclic ADP-ribose (cADPR) [4].

The ADPRibase-Mn substrates ADP-ribose and cADPR are intracellular signal molecules with known effects on TRPM2 ion channels [5–9] or, in the case of cADPR, on ryanodine receptors [10–12]. Both are putatively formed, in mammals, from NAD^+^ by CD38 and Bst-1/CD157 (reviewed in [13]). The calcium regulator cADPR is an analog of ADP-ribose in which adenine, besides the standard N9-glycosydic linkage with the C1´ atom of the adenosine moiety, establishes also a N1-glycosydic linkage with the C1´´ atom of the other (‘northern’) ribose, thus forming a macrocyclic structure [12]. In some animals, like *Aplysia*, cADPR synthesis is catalyzed from NAD^+^ by an ADP-ribosyl cyclase specifically acting as such [14–16], although in mammals it is synthesized by a quantitatively minor alternative pathway of the NAD glycohydrolases CD38 and Bst-1/CD157, that mainly hydrolyze NAD^+^ to ADP-ribose. [17–24]. In mammals, the turnover of cADPR signaling is exerted also by CD38 and Bst-1/CD157, which convert cADPR to ADP-ribose by hydrolysis of the N^1^-glycosidic linkage [17–19, 21, 23]. The diphosphate group of cADPR is resistant to phosphohydrolases of broad specificity [4, 18, 25], like snake venom phosphodiesterase which otherwise hydrolyzes a large variety of 5´-nucleotide phosphodiester or phosphoanhydride derivatives including e.g. ADP-ribose [26–28]. It is thus remarkable that rat ADPRibase-Mn acts as cADPR phosphohydrolase with low but significant efficiency (about 100-fold lower than the activity on ADP-ribose), yielding N^1^-(5-phosphoribosyl)-AMP as the product. This could hypothetically represent a novel route for cADPR turnover [4]. Interestingly, cADPR and its product of hydrolysis by ADPRibase-Mn, N^1^-(5-phosphoribosyl)-AMP, have been identified in a metabolomic profile of human erythrocytes infected with *Plasmodium falciparum* [29]. Also interestingly, in the search for pharmacologically-active cADPR analogs [30–33], the previously overlooked possibility of cADPR phosphohydrolysis is now being taken into account [34–36].

The taxonomic distribution of ADPRibase-Mn is intriguing, as it has been reported to be restricted, among eukaryotes, to vertebrates and plants [2]. Furthermore, biochemical and in-silico expression data indicate that ADPRibase-Mn is preferentially expressed in immune cells of rodents [2, 37]. Related to this pattern, it is worth recalling that CD38 and Bst-1/CD157, the proteins that form ADP-ribose and cADPR, have a regulatory role at the interface between the innate and the adaptative immune systems [38–41]. In this field there are still some uncompletely solved issues. One is the so-called topological paradox posed by the location of mammalian proteins forming ADP-ribose and cADPR in the outer surface of plasma membrane, against the cytosolic action of these compounds. Several explanatory mechanisms for this apparent paradox have been advanced [12, 42–45]. The intracellular termination of these two signals and of their effects is also worth of consideration. In addition to ADPRibase-Mn, two mammalian enzymes of the Nudix superfamily are known that can efficiently hydrolyze ADP-ribose [1, 46–49]. However, for the turnover of cADPR, as stated above, only its conversion to ADP-ribose by the glycohydrolytic activity of CD38 and Bst-1/CD157, i.e. the same membrane proteins responsible for its synthesis, is generally mentioned. Therefore, it remains to be seen whether ADPRibase-Mn could be involved in this regulatory network with a role in the turnover of cADPR [4].

ADPRibase-Mn proteins belong to the metallo-dependent phosphatases (MDP) SCOP2 superfamily, within which they form a structural family of their own (ADPRibase-Mn-like; ID 4002589) [50]. In the CATH classification, ADPRibase-Mn proteins form also a unique functional family within the structural cluster SC:2 of superfamily 3.60.21.10 [51]. The only ADPRibase-Mn enzymes that have been biochemically studied are those from rat [1, 2, 4] and zebrafish [52]. The structure of zebrafish ADPRibase-Mn in complex with P_i_ is deposited in Protein Data Bank (PDB ID 2NXF), and it has been used as template to construct homology models of murine and human proteins [53]. Like other MDPs, the putative active site of ADPRibase-Mn contains a dimetallic center, possibly Fe/Mn, as indicated for the zebrafish ADPRibase-Mn (http://www.uwstructuralgenomics.org/gallery/2nxf.pdf [54]).

Concerning substrate specificity, ADPRibase-Mn enzymes are Mn^2+^-dependent ADP-ribose/CDP-alcohol diphosphatases that hydrolyze the phosphoanhydride linkage of ADP-ribose and, somewhat less efficiently, of CDP-choline, CDP-glycerol and CDP-ethanolamine. The catalytic efficiency of ADP-ribose hydrolysis, depending on the enzyme source (rat or zebrafish), is 4–60-fold higher than that of CDP-alcohol hydrolysis, and it is a reflection of the low *K*_m_ value for ADP-ribose, in spite of the higher *k*_cat_ shown for CDP-alcohols in every case [2, 52]. Besides the diphosphatase activities on ADP-ribose and CDP-alcohols, ADPRibase-Mn enzymes display also activity on ADP and 2´,3´-cAMP with similar or lower efficiency than on CDP-alcohols, and as stated above the rat enzyme is able to hydrolyze the phosphoanhydride linkage of cADPR. The overall specificity of ADPRibase-Mn enzymes is highlighted by their lack of significant activity towards ADP-glucose, UDP-glucose, CDP-glucose, CDP, CMP, AMP or 3´,5´-cAMP [1, 2, 52]. In contrast to the rat enzyme, zebrafish ADPRibase-Mn does not hydrolyze cADPR significantly, being in this concern at least 150-fold less efficient than the rat enzyme, while both of them are equally efficient on ADP-ribose [52]. Therefore, it remains to be established whether the cADPR phosphohydrolase activity is a peculiarity of rat ADPRibase-Mn or may be shared at least by enzymes from other mammalian sources.

The molecular basis of ADPRibase-Mn specificity in terms of interactions between substrates and protein has not been so far investigated by mutagenesis. In the present work, we have aimed to understand the catalytic behavior of human ADPRibase-Mn by performing an extensive mutagenesis study based on substrate docking simulations. Important questions are answered on the molecular basis of the enzyme preference for ADP-ribose and the cADPR phosphohydrolase activity of mammalian members of the ADPRibase-Mn-like family. Interestingly, a triple mutant of human ADPRibase-Mn acts preferentially as cADPR phosphohydrolase, due to an increased catalytic efficiency on cADPR and decreased efficiencies on other substrates.

## Materials and Methods

### Materials

The sources of the products used, including cADPR purified from the commercial preparation, were as described elsewhere [2, 4, 52].

### Cloning and sequencing of human ADPRibase-Mn cDNA

The open reading frame (ORF) of human ADPRibase-Mn was cloned from human liver cDNA (Marathon-Ready cDNA, Clontech) by PCR. The primers were based on the *ADPRM* mRNA accession no. NM_020233, which codes for the hypothetical 342-aa protein NP_064618, that is 86% and 51% identical with the biochemically-studied ADPRibase-Mn enzymes from rat (ABW03224 [2, 4]) and zebrafish (2NXF_A [52]), respectively. The sequence of the forward primer was CTAGCGTCGACATGGATGATAAACCCAACCCTGAAGCCC, which includes a SalI site (single underline) preceding the ORF initiation codon (double underline); and that of the reverse primer was CAGGTGCGGCCGCCTAACAATGGAATGCTCTTTCTTTC, which includes a NotI site (single underline) after the stop codon (double underline). The amplicon was first cloned in pGEM-T Easy vector by T/A ligation and, after identification of a clone with the expected insert, it was excised and subcloned in frame with the glutathione S-transferase (GST) tag of the pGEX-6P-3 vector cut with NotI and SalI, yielding plasmid pGEX-6P-3-hADPRM. The 1029-nt sequence of the human ORF insert was confirmed by double-strand sequencing (Servicio de Genómica, Instituto de Investigaciones Biomédicas Alberto Sols, Consejo Superior de Investigaciones Científicas-Universidad Autónoma de Madrid, Madrid) and deposited in GenBank with accession number KF880964. It coincided with the ORF included in mRNA NM_020233, except for the silent substitutions C18T and A1017C due to primer design. The theoretical translation of the cloned ORF (accession number AHG56452) was thus fully identical to the hypothetical 342-aa protein NP_064618.

### Site-directed mutagenesis

Point mutants were constructed following the *QuikChange* protocol (Stratagene) using mutagenic primer pairs and pGEX-6P-3-hADPRM as template. The double mutants were constructed starting from pGEX-6P-3-F37A-hADPRM, and the triple one from pGEX-6P-3-F37A+L196F-hADPRM. The forward mutagenic primers are shown in S1 Table; the reverse primers were the exact reverse complement of those. The correctness of all the mutants was confirmed by double-strand sequencing of the complete coding sequence as above.

### Expression and purification of human ADPRibase-Mn protein and its mutant forms

The wild type and mutant proteins were expressed as fusions with glutathione S-transferase (GST) from pGEX-6P-3-hADPRM or the corresponding mutated plasmid. The recombinant proteins were purified by affinity chromatography on glutathione-Sepharose 4B (GE Healthcare), and separated from the GST tag by specific proteolysis with the PreScission protease [2]. This yielded each protein with an 11-amino acid N-terminal extension (GPLGSPNSRVD). Protein concentration was assayed in the final preparations according to Bradford [55] and the purity was estimated by SDS-PAGE stained with Coomassie Blue followed by quantitation by image analysis with the GelAnalyzer 2010 software (http://www.gelanalyzer.com; last access on December 2, 2014). In all cases, a single major protein band of ≈40.5 kDa, the approximate expected size for recombinant ADPRibase-Mn and its mutants, was observed. It accounted in the different preparations for 52%–79% of protein in the gel lanes. The estimations of purity for each protein are summarized in S2 Table. Since the wild type ADPRibase-Mn was 70% pure, the purity ratios of the mutants versus the wild type ranged 0.74–1.13. So the minor differences of purity between some of the mutants and the wild type do not affect to an important extent the estimation of the shifts of *k*_cat_ and *k*_cat_/*K*_m_ elicited by the mutations.

### Enzyme assays

All the enzyme assays and kinetic studies were as described elsewhere, and were linear with time and ADPRibase-Mn amount [4, 52]. In summary, phosphohydrolytic reactions were studied by measuring colorimetrically the P_i_ liberated by alkaline phosphatase from the reaction products, with the exceptions of the phosphohydrolytic reactions of CDP, CMP, ADP and AMP, for which the P_i_ liberated was measured without including alkaline phosphatase in the assay, and of cADPR phosphohydrolysis which was studied by following the accumulation of *N* ^1^-(5-phosphoribosyl)-AMP by HPLC (see below). Under the standard conditions, the assays were conducted at 37°C, pH 7.5, and in the presence of 5 mM MnCl_2_, unless otherwise stated. Saturation analysis was conducted from initial-rate assays. The *k*_cat_ and *K*_m_ values were obtained by nonlinear regression of the Michaelis-Menten equation to the experimental data points using the Solver tool of the Microsoft Excel 2004 for the Mac version 11.0. In cases where separate *k*_cat_ and *K*_m_ values could not be estimated, the *k*_cat_/*K*_m_ ratio (catalytic efficiency) was calculated from initial-rate assays at low substrate when velocity is proportional to substrate concentration (*S*) and the enzyme is largely unbound to substrate. Under these conditions *k*_cat_/*K*_m_ = *v/*(*E S*), *E* being the total enzyme concentration [56].

### High performance liquid chromatography

A HP-1100 chromatograph (Hewlett-Packard) was used with a manual injector equipped with a 20-μl injection loop and a diode array detector. The analyses were performed by ion-pair reverse-phase HPLC in a 15 cm x 0.4 cm octadecylsilica column (Kromasil 100; Teknokroma, Sant Cugat del Vallés, Barcelona, Spain) with a 1 cm x 0.4 cm pre-column of the same material. The chromatographic conditions for analyses of enzyme reaction products were as described for the hydrolysis of cADPR [4] or 2´,3´-cAMP [52]. Chromatogram analyses were performed with the HP ChemStation software.

### Sequence analyses

Searches for eukaryotic ADPRibase-Mn homologs were done by BlastP analyses [57], using the human protein as query, against NCBI_nr or NCBI_RefSeq databases (last access on July 7, 2014) [58]. The former was used to test for the absence of homologous proteins in specific taxonomic groups; the latter was mainly used to retrieve a set of representative homologous proteins for sequence alignment. This set of proteins was prepared such that (i) only one protein per species was included (the one with lower E value when compared with human ADPRibase-Mn); (ii) proteins with an identity higher than 95% with any other already selected were discarded (in order to avoid overrepresentation of particular taxonomic groups); (iii) proteins with less than an 80% of query coverage or without the complete MDP-superfamily motif (DX[H/X]-X_n_-GDXX[D/X]-X_n_-GNH[D/E]-X_n_-[G/X]H-X_n_-GHX[H/X] [59–63]) were also discarded. Protein alignments were performed with Clustal Omega [64] using default parameters and analyzed and colored with ESPript [65].

### Homology model of human ADPRibase-Mn

The coordinates of the homology model of human ADPRibase-Mn were downloaded on July 24, 2011 from the SWISS-MODEL repository [53]. This model is based on the known structure of zebrafish ADPRibase-Mn (Protein Data Bank ID 2NXF), with which the human protein has 66% similarity, including 51% identity. The template and the downloaded model lacked the fourteen N-terminal and the six C-terminal residues, so to obtain a complete model they were built with Modeller [66]. The coordinates for the two metal ions and the water molecule coordinated with them were taken from the structure of zebrafish ADPRibase-Mn.

### Substrate docking

The model of ADPRibase-Mn and its ligands were prepared for docking as described [2, 4, 52]: the ligand coordinates were generated with Marvin (Chemaxon), the receptor and the ligands were prepared for docking with AutoDock Tools [67] and docking calculations were run with AutoDock [68]. Two hundred conformations were obtained for each substrate docked, except that 1000 conformations were obtained for cADPR. For selection of the best docked conformations, it was hypothesized that the metal-bridging water could act as the attacking nucleophile over the P atom participating in the substrate scissile bond, like in the mechanisms of other phosphoesterases [61, 69]. For ADP-ribose, CDP-choline and cADPR, the best conformation was taken as the one with the lowest energy level among those in which the angle formed by the O-to-P attack line and the scissile P-O bond was ≥170°. For 2´,3´-cAMP, which posed in two different orientations favoring either 3´-AMP or 2´-AMP as product, the conformation chosen had the largest attack angle among those showing the orientation favorable to 3´-AMP formation. In agreement with the above mentioned hypothesis, one of the substrate P atoms was at short distance (< 3 Å) of the metal-bridging water oxygen in all the selected conformations. The coordinates of the four docking complexes with the model structure of human ADPRibase-Mn are included in S1–S4 Dataset.

### Molecular dynamics simulations

The complex of human ADPRibase-Mn with ADP-ribose obtained by docking was the starting structure for the simulation of molecular dynamics (MD) of the enzyme-substrate complex and the free enzyme (after ADP-ribose removal). Geometry optimizations and MD simulations were performed with the GROMACS package (version 4.0.5) [70] in combination with the GROMOS 53A6 force field [71]. For the simulation of the enzyme-substrate complex, force field parameters for ADP-ribose were assigned based on analogous functional groups already present in the force field library. Each system was immersed in a cubic box of SPC water molecules [72] and 50 mM NaCl. The minimum distance between the protein and the walls of the periodic box was 10 Å. The energy of each system was minimized with 2000–2500 steps of steepest descent algorithm. Equilibration was carried out in three steps: first, 20 ps of constant volume equilibration were performed at 300 K with position restraints on the protein; second, 200 ps of constant pressure equilibration with position restraints on the protein; third, 100 ps of constant pressure equilibration without position restraints. Production runs were started at this point. Bond lengths of protein (and ligand) were constrained with LINCS [73, 74] and those of water with SETTLE [75], allowing for a 2-fs time step. The non-bonded interactions were treated with a triple-range schema with cut-off radii of 8 Å and 14 Å. The short-range van der Waals and electrostatic interactions were evaluated every time step from a pairlist that was generated every five steps. Medium-range van der Waals and electrostatic interactions (between 8 Å and 14 Å) were computed every fifth step and kept constant between pairlist updates. Long-range electrostatic interactions outside the longer cut-off were accounted for by a reaction field with a relative dielectric permittivity of 66. The temperature (300 K) and pressure (1 atm) were kept constant using the Berendsen thermostat and barostat [76] with τ_T_ = 0.1 ps and _P_ = 0.5 ps, respectively. Coordinates were saved every 1 ps. To help maintaining the geometry of the dimetallic center, the distances between the metal ions and the coordinated water molecule were constrained to their initial distances.

## Results and Discussion

### Catalytic specificity of human ADPRibase-Mn including cADPR phosphohydrolase activity

The catalytic specificity of the recombinant protein was as expected for a typical ADPRibase-Mn [1, 52]. When phosphohydrolytic activities were assayed in the presence of 5 mM Mn^2+^, at a fixed 500 μM substrate concentration, ADP-ribose was hydrolyzed at the highest rate. The CDP-alcohols (CDP-choline, CDP-ethanolamine and CDP-glycerol) followed near at 70%–90% of the ADP-ribose rate. Somewhat slower (40%) was the hydrolysis of 2´,3´-cAMP, and even slower the reactions of ADP and cADPR. ADP-glucose, UDP-glucose, CDP, CMP, AMP, 3´,5´-cAMP were not hydrolyzed at detectable rates (< 0.1%). The results are summarized in S3 Table.

The reaction product of 2´,3´-cAMP hydrolysis, assayed by HPLC, was mainly 3´-AMP, which corresponds to the hydrolysis of the ester linkage on the 2´-O side of the phosphodiester, although a minor proportion of 2´-AMP was also formed (< 3% of 3´-AMP), like with zebrafish ADPRibase-Mn [52]. The product of cADPR hydrolysis, also assayed by HPLC, was *N* ^1^-(5-phosphoribosyl)-AMP, which corresponds to the hydrolysis of the phosphoanhydride linkage of cADPR like with rat ADPRibase-Mn [4].

The responses of activity to varying pH or Mn^2+^ concentration were studied with ADP-ribose, CDP-choline and 2´,3´-cAMP. The hydrolysis of ADP-ribose, assayed at pH 6.0–10.5, showed a symmetrical bell-shaped profile with maximal activity at pH 8.5, whereas the hydrolysis of the other substrates showed a rather flat profile at pH 6.0–8.5 followed by a steep decrease at higher pH values (S1 Fig.). This behavior is similar to that shown by both the rat and the zebrafish ADPRibase-Mn [2, 52]. The different pH-activity profiles seem to indicate that the hydrolysis of ADP-ribose, but not the other substrates, depend on the dissociation of an acidic group with apparent p*K*_a_ between 7 and 8. The responses of activity to Mn^2+^ were near complete at about 10 μM metal and remained essentially without change up to 5 mM (S2 Fig.), similarly to the rat ADPRibase-Mn [1, 2].

Saturation kinetics at varying concentrations of substrate, with Mn^2+^ concentration fixed at 5 mM and at pH 7.5, was studied with ADP-ribose, CDP-choline, 2´,3´-cAMP and cADPR. The best substrate in terms of catalytic efficiency (*k*_cat_/*K*_m_) was ADP-ribose due to its lower *K*_m_ value, followed by CDP-choline, 2´,3´-cAMP and cADPR (Table 1). The phosphoanhydride linkage of cADPR was hydrolyzed with the same catalytic efficiency as the rat enzyme [4], i.e. about 4000 M^-1^s^-1^, which is at least 150-fold higher than the efficiency of the zebrafish enzyme [52]. The difference was due to the much higher *k*_cat_ values of the mammalian enzymes for this substrate, compared to the enzyme from the fish. This confirmed that a relatively robust cADPR phosphohydrolase activity may be a general feature of mammalian ADPRibase-Mn. In this concern, and as earlier pointed out, a ratio of efficiencies for ADP-ribose/cADPR hydrolysis near 100 (Table 1 and [4]) is near the ratio of ADP-ribose/cADPR synthesis from NAD by CD38, the major cADPR-forming enzyme in mammals [15, 18, 20, 77]. Therefore, the potential of ADPRibase-Mn for cADPR turnover may be relevant.

**Table 1:** Kinetic parameters of human ADPRibase-Mn and mutants.

Mutations	Substrate	*k*_cat_		*K*_m_		*k*_cat_/*K*_m_	
		*s* ^*-1*^	*Fold change*	*µM*	*Fold change*	*M* ^*-1*^ *s* ^*-1*^	*Fold change*
Wild type	ADP-ribose	35 ± 11	–	60 ± 8	–	590000 ± 160000	–
	CDP-choline	50 ± 4	–	350 ± 60	–	150000 ± 30000	–
	2´,3´-cAMP	60 ± 13	–	2400 ± 300	–	25000 ± 6000	–
	cADPR	3.2 ± 0.2	–	780 ± 200	–	4000 ± 1000	–
Q27H	ADP-ribose	10 ± 3	↓4	200 ± 14	↑3	52000 ± 10000	↓11
	CDP-choline	39 ± 12	≈	2700 ± 200	↑8	14000 ± 3000	↓11
	2´,3´-cAMP	4.3 ± 1.1	↓14	2300 ± 130	≈	1900 ± 400	↓13
	cADPR	na	na	na	na	150 ± 20	↓27
F37A	ADP-ribose	14 ± 3	↓3	1150 ± 80	↑19	12000 ± 2000	↓50
	CDP-choline	33 ± 7	↓1.5	970 ± 140	↑3	34000 ± 3000	↓4
	2´,3´-cAMP	36 ± 6	↓1.7	5100 ± 300	↑2	7000 ± 700	↓4
	cADPR	na	na	na	na	1300 ± 200	↓3
F37Y	ADP-ribose	50 ± 11	≈	77 ± 10	≈	650000 ± 170000	≈
	CDP-choline	76 ± 23	≈	430 ± 50	≈	180000 ± 60000	≈
	2´,3´-cAMP	70 ± 18	≈	2500 ± 180	≈	28000 ± 5000	≈
	cADPR	na	na	na	na	4100 ± 100	≈
R43A	ADP-ribose	0.003 ± 0.003	↓12000	115 ± 30	↑1.9	30 ± 30	↓20000
	CDP-choline	0.025 ± 0.004	↓2000	9000 ± 8000	↑26	6 ± 6	↓25000
	2´,3´-cAMP	0.95 ± 0.14	↓60	7600 ± 1900	↑3	130 ± 20	↓200
	cADPR	na	na	na	na	0.1 ± 0.1	↓40000
N110A	ADP-ribose	4.3 ± 0.6	↓8	290 ± 50	↑5	15000 ± 200	↓40
	CDP-choline	5.7 ± 1.4	↓9	4000 ± 1100	↑11	1500 ± 400	↓100
	2´,3´-cAMP	0.11 ± 0.01	↓550	1050 ± 60	↓2	100 ± 10	↓250
	cADPR	na	na	na	na	130 ± 30	↓31
H111A	ADP-ribose	0.47 ± 0.08	↓75	140 ± 14	↑2	3000 ± 200	↓200
	CDP-choline	2.2 ± 0.2	↓23	7500 ± 700	↑21	300 ± 30	↓500
	2´,3´-cAMP	7.4 ± 2.9	↓8	1250 ± 450	↓2	6000 ± 400	↓4
	cADPR	na	na	na	na	70 ± 6	↓60
H111N	ADP-ribose	2.3 ± 0.2	↓15	78 ± 5	≈	29000 ± 4000	↓20
	CDP-choline	5.3 ± 1.0	↓9	2700 ± 500	↑8	2000 ± 400	↓75
	2´,3´-cAMP	34 ± 6	↓1.8	1800 ± 450	≈	19000 ± 2000	≈
	cADPR	na	na	na	na	290 ± 40	↓14
L196A	ADP-ribose	16 ± 4	↓2	130 ± 5	↑2	120000 ± 30000	↓5
	CDP-choline	29 ± 13	↓1.7	470 ± 90	≈	62000 ± 20000	↓2
	2´,3´-cAMP	30 ± 10	↓2	3400 ± 500	≈	9000 ± 4000	↓3
	cADPR	na	na	na	na	900 ± 100	↓4
F210A	ADP-ribose	19 ± 3	↓1.8	2100 ± 100	↑34	9000 ± 1000	↓70
	CDP-choline	50 ± 12	≈	11000 ± 2600	↑32	4000 ± 100	↓40
	2´,3´-cAMP	2.7 ± 0.2	↓22	7600 ± 1700	↑3	400 ± 10	↓60
	cADPR	na	na	na	na	8 ± 1	↓500
C253A	ADP-ribose	97 ± 8	↑3	94 ± 6	↑1.6	1000000 ± 100000	↑1.7
	CDP-choline	79 ± 7	↑1.6	500 ± 70	≈	160000 ± 20000	≈
	2´,3´-cAMP	83 ± 5	≈	2600 ± 250	≈	32000 ± 2000	≈
	cADPR	8.9 ± 0.8	↑3	200 ± 40	↓4	44000 ± 9000	↑11
F37A+L196A	ADP-ribose	7 ± 2	↓5	1150 ± 500	↑19	6000 ± 2000	↓100
	CDP-choline	25 ± 5	↓2	1250 ± 250	↑4	21000 ± 8000	↓7
	2´,3´-cAMP	19 ± 2	↓3	4800 ± 900	↑2	4000 ± 1000	↓6
	cADPR	na	na	na	na	900 ± 100	↓4
F37A+L196F	ADP-ribose	8 ± 1	↓4	1600 ± 100	↑26	4800 ± 200	↓120
	CDP-choline	26 ± 3	↓1.9	1500 ± 250	↑4	17000 ± 3000	↓9
	2´,3´-cAMP	28 ± 3	↓2	7100 ± 650	↑3	3900 ± 400	↓6
	cADPR	na	na	na	na	1600 ± 200	↓3
F37A+L196F+C253A	ADP-ribose	13 ± 1	↓3	1200 ± 230	↑20	11000 ± 1700	↓50
	CDP-choline	37 ± 5	≈	1700 ± 120	↑5	22000 ± 2600	↓7
	2´,3´-cAMP	20 ± 3	↓3	5200 ± 1000	↑2	4000 ± 800	↓6
	cADPR	16 ± 3	↑5	460 ± 120	↓1.7	35500 ± 4300	↑9

The values with S.D. are averages of at least three experiments. Fold change was calculated with respect to the parameters of the wild type. ≈, change < 1.5 fold; na, not assayed (in these cases *k*_cat_/*K*_m_ was calculated from initial-rate assays at low substrate when velocity was proportional to substrate concentration; see under Materials and Methods).

### Reexamination of the taxonomic distribution of the ADPRibase-Mn-like family

It has been reported that, among animals, the ADPRibase-Mn-like protein family has a phylogenetic distribution restricted to vertebrates, although it is present also in plants and algae [2]. This distribution was here reexamined to account for the considerable increase in database size since that report, and to test the degree of conservation of important residues revealed by the present study (see below). The results confirmed essentially the above-mentioned distribution with minor but perhaps important exceptions. ADPRibase-Mn proteins appeared widespread in vertebrates (possibly also in other Chordata and in Hemichordata) and plants, were absent from fungi, and irregularly distributed among protists. Concerning invertebrates, significant ADPRibase-Mn relatives were absent from most taxa, which included those with some organism(s) with well characterized genomes, like Arthropoda, Echinodermata and Nematoda. However, there was the important exception of Mollusca, where ADPRibase-Mn-like proteins seem to occur in Gastropoda (*Aplysia californica* and *Lottia gigantea*) and Bivalvia (*Crassostrea gigas*). A significant homolog was also found in Annelida (*Helobdella robusta*). Of course, it remains to be seen whether these proteins have ADPRibase-Mn enzyme activities. A summary of the current view of this protein family is shown in S3 Fig. under the form of a sequence alignment.

### Identification of amino acids interacting with ADP-ribose in the model structure of the enzyme-substrate complex and their relationship to defined regions of human ADPRibase-Mn

**Fig 1:**
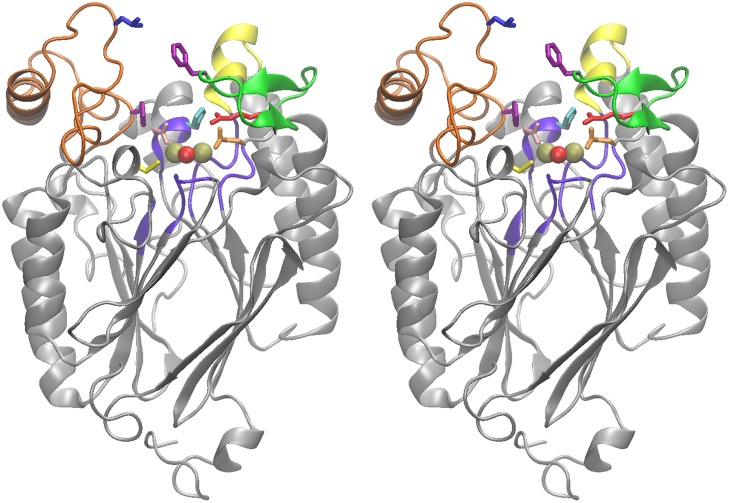
Stereogram of the model structure of human ADPRibase-Mn. Schematic model without substrate, showing metals (golden spheres), metal-bridging water (red sphere), the five short consensus regions of the MDP-superfamily motif (violet), and the ADPRibase-Mn unique regions s2s3 (green), h2 (yellow), and h7h8 (orange). The amino acids chosen for mutagenesis are highlighted and colored by residue: Gln^27^ (orange), Phe^37^ (magenta, in s2s3), Arg^43^ (red), Asn^110^ (pink), His^111^ (cyan), Leu^196^ (blue), Phe^210^ (magenta; in h7h8) and Cys^253^ (yellow). The model is based, as indicated in Materials and Methods, in the structure of the zebrafish homolog (PDB ID 2NXF).

**Fig 2:**
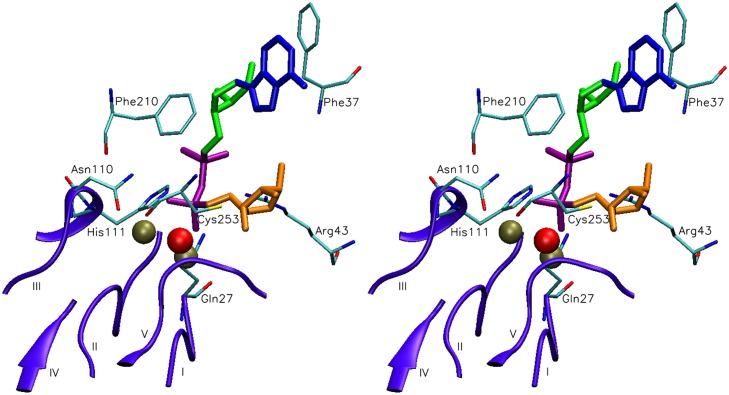
Stereogram of ADP-ribose docked to the active site of the model structure of human ADPRibase-Mn: identification of interacting amino acids. Besides ADP-ribose, the model shows the dimetallic center bound to the five short consensus regions of the MDP-superfamily motif (I-V, violet), and the amino acids identified by their interaction with docked ADP-ribose (see the main text for details). This set of hypothetic interactions was the starting point for the mutagenesis experiments. The conformation shown in the figure was the starting structure for the molecular dynamics simulation of the enzyme-substrate complex (Fig. 3).

The ADPRibase-Mn family contains the disperse motif of the MDP superfamily that responds to the description DX[H/X]-X_n_-GDXX[D/X]-X_n_-GNH[D/E]-X_n_-[G/X]H-X_n_-GHX[H/X], i.e. five short consensus regions separated by long tracts (X_n_) of nonconserved sequence that, however, form a well conserved structure. The five short conserved regions are marked in Fig. 1, Fig. 2 and S3 Fig. They are part of loops located at the end of β strands and contains the amino acids that form the dinuclear center by coordination to the metals [59–63]. Other than the MDP superfamily motif, very little sequence conservation is found when the ADPRibase-Mn-like family is compared to the other eleven MDP families classified in SCOP2. However, there is ample and strong conservation of structure throughout the superfamily. Against this background, three structural elements stand out as unique to the ADPRibase-Mn-like family: using the numbering of zebrafish protein sequence, they correspond approximately to amino acids 20–35, 65–70 and 150–195 [52]. These will be here named, respectively, regions s2s3, h2, and h7h8, according to the secondary structure elements (s, strand; h, helix) of zebrafish ADPRibase-Mn that they contain. These unique regions delimit the entrance to the active site of zebrafish ADPRibase-Mn and of the homology models for which it served as template. They are present throughout the ADPRibase-Mn-like family with a significant degree of sequence conservation (S3 Fig.), and they are candidates to contain substrate specificity determinants.

**Fig 3:**
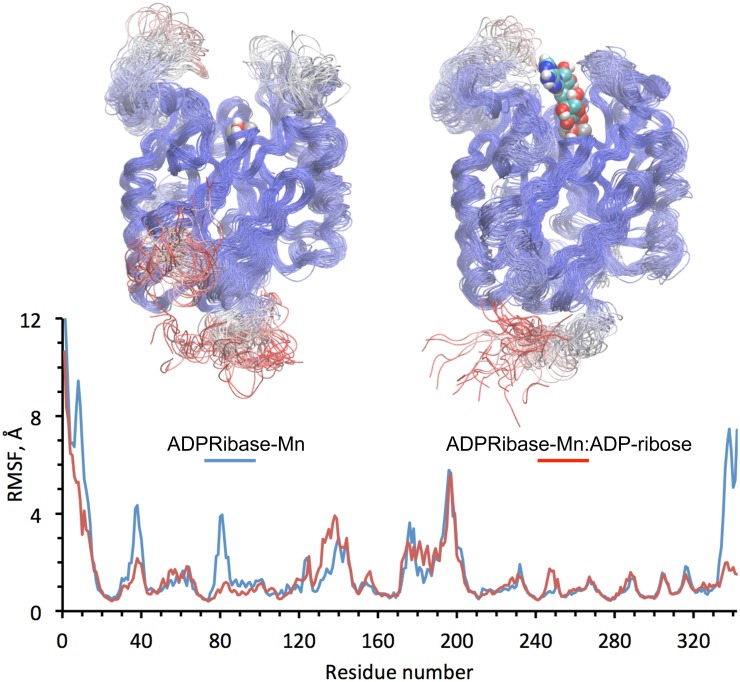
Molecular dynamics simulation of the complex of modeled human ADPRibase-Mn with ADP-ribose, and its comparison to the substrate-free enzyme. Simulations of 40 ns were implemented as described under Materials and Methods. The upper protein models show the overlap of 40 snapshots taken at 1-ns intervals, with the protein backbone represented as a thin tube colored by mobility (red, maximal mobility; blue minimal mobility). The left-hand side and the right-hand side models contain the dimetallic center and the bridging water, and the right-hand side one contains also docked ADP-ribose colored by element. These ligands are shown in the positions occupied at the beginning of the simulations. The lower graph shows the mobility of the Cα atoms of the protein backbone quantitated as RMSF values.

To simulate the binding of substrates to human ADPRibase-Mn, a homology model, based on the known structure of zebrafish ADPRibase-Mn, was taken from a public repository and completed as described under Materials and Methods. It displayed a deep pocket with a dinuclear center in the bottom and a metal-bridging water. Around the pocket entrance stood the unique regions s2s3, h2, and h7h8 (Fig. 1). ADP-ribose, cADPR, CDP-choline and 2´,3´-cAMP were independently docked to this site (S4 Fig.).

The conformation of ADP-ribose docked to the model of human ADPRibase-Mn is shown in Fig. 2, which displays also the amino acids that interact with ADP-ribose as identified by analysis with VMD. This included amino acids belonging (i) to the ADPRibase-Mn-unique regions s2s3 (Phe^37^ and Arg^43^) or h7h8 (Phe^210^), (ii) to the MDP superfamily motif (Gln^27^, Asn^110^ and His^111^), or (iii) to the short h10 helix (Cys^253^). These seven amino acids interact also with ADP-ribose docked to a homology model of rat ADPRibase-Mn [2]. Their locations in human ADPRibase-Mn are highlighted in Fig. 1, as it is the position of Leu^196^ within h7h8 because, although it did not interact with ADP-ribose docked to human ADPRibase-Mn, it does so with the substrate docked to rat ADPRibase-Mn [2]. These eight amino acids of the human enzyme were subjected to mutagenesis.

### Point mutations in human ADPRibase-Mn and effects on kinetic parameters

Most of the mutations implemented were intended to inactivate a potential effect of the interacting amino acid, and the native residues were changed to alanine (some control mutations were performed to non-alanine residues). The only exception was the mutation of Gln^27^, aimed to test the effects of its substitution by a histidine residue, which is present in other MDP proteins.

The effects of the mutations were tested by kinetic assay of *k*_cat_, *K*_m_ and *k*_cat_/*K*_m_ with ADP-ribose, CDP-choline, 2´,3´-cAMP and cADPR. With the latter substrate, in many cases, rather than measuring *k*_cat_ and *K*_m_ from saturation curves, the catalytic efficiency parameter *k*_cat_/*K*_m_ was directly estimated under conditions of rate linearity *versus* cADPR concentration, i.e. at cADPR concentrations well below the *K*_m_ value [4, 52, 56]. The results are summarized in Table 1.

The majority of the mutations to alanine of the amino acids interacting with ADP-ribose diminished the catalytic efficiency for this substrate to different extents. Sometimes, but not always, roughly similar decreases of efficiency were observed with the other substrates. However, an important exception was the absence of negative effect of mutating Cys^253^ which, as stated above, was the only residue unrelated to the disperse motif of the MDP superfamily and to the regions unique to the ADPRibase-Mn-like family. Actually, the C253A mutant displayed a ten-fold increased efficiency over cADPR, with no or modest effect on the other substrates. The meaning of these and other results of the mutagenesis study, which included a few double and one triple mutant, is presented and discussed below in detail.

### Roles of amino acids belonging to the disperse motif of the MDP superfamily: Gln^27^, Asn^110^ and His^111^

Three of the amino acids that have been mutated in human ADPRibase-Mn belong to two of the short consensus regions of the MDP superfamily motif.

Gln^27^ occupies the third position of the first consensus region (DX[H/X]), that in the ADPRibase-Mn-like family is conserved as D[V/I]Q with very few exceptions (see S3 Fig.). In the docking model with ADP-ribose, the carbonyl group of Gln^27^ side chain is coordinated with one of the metal ions (metal 1), and the amide NH_2_ is near to form a hydrogen bond with one of the oxygens of the β phosphate of ADP-ribose. We chose to implement the Q27H mutation that does not eliminate the possibility of side-chain interactions and converts the D[V/I]Q region to D[V/I]H that is the more general consensus of the MDP superfamily [59–63]. The mutation did not change the relative preference of the enzyme for the four substrates considered as it reduced to the same extent (≈11–13-fold reduction) the catalytic efficiency of the hydrolysis of ADP-ribose, CDP-choline or 2´,3´-cAMP, and only somewhat more strongly (≈27-fold reduction) the hydrolysis of cADPR (see Table 1). However, the effects on *k*_cat_ and *K*_m_ differed among the three first substrates. The *k*_cat_ value diminished 4 fold for ADP-ribose, was unchanged for CDP-choline and diminished 14 fold for 2´,3´-cAMP. The *K*_m_ value augmented 3 fold for ADP-ribose, 8 fold for CDP-choline and did not change for 2´,3´-cAMP. There was no data available on *k*_cat_ and *K*_m_ for cADPR. In summary, for ADP-ribose, the Q27H substitution elicited modest negative effects of similar magnitude in catalysis and in substrate binding. For CDP-choline, the substitution did not affect catalysis, only binding. On the contrary, for 2´,3´-cAMP, the substitution affected catalysis not binding, perhaps because the larger size of histidine made less likely for this substrate to adopt a favorable orientation of the scissile P-O bond with respect to the water nucleophile.

Asn^110^ is part of the GNH[D/E] consensus region as an invariant residue (S3 Fig.). In the docking model with ADP-ribose, the side-chain carbonyl of Asn^110^ is coordinated with one of the metal ions (metal 2) and its amide NH_2_ is donor in a hydrogen bond with one of the oxygens of the phosphate of ADP-ribose. We chose to implement in this case a N110A mutation that might affect both the coordination sphere of the dinuclear center and the binding of substrate. The effects of this substitution on kinetic parameters (Table 1) showed a complex response that partly recalled the effects of the Q27H mutation but with a few important differences. First, with the N110A substitution the reduction of catalytic efficiency was stronger (100–250 fold) for the hydrolysis of CDP-choline or 2´,3´-cAMP than for the hydrolysis of ADP-ribose or cADPR (30–40 fold). The decrease of *k*_cat_ value was stronger for the hydrolysis of 2´,3´-cAMP which was 550 fold smaller than the wild type *k*_cat_. The changes of *K*_m_ were little different to those elicited by the Q27H substitution. The results of this mutation are difficult to interpret as, besides the possible effects on the metal coordination sphere and on the binding of substrate, the proximity of Asn^110^ to His^111^ could affect the interaction of the latter with substrates (see below).

His^111^ is also an invariant residue of the GNH[D/E] consensus like Asn^110^ (S3 Fig.), and in non-ADPRibase-Mn MDPs it has been found important in catalysis but not involved in metal coordination [78–81]. In ADPRibase-Mn, the GNH[D/E] histidine residue is neither involved in metal coordination, but is at hydrogen bond distance of the phosphoanhydride oxygen of diphosphate substrates or of the 2´-phosphoester of 2´,3´-cAMP (S4 Fig.). This has been observed also in docking complexes of zebrafish ADPRibase-Mn [52]. We implemented two His^111^ mutants: H111A and H111N. The first one caused a marked efficiency decrease with all substrates except 2´,3´-cAMP, which was little affected. The same response pattern was produced by the equivalent mutation in zebrafish ADPRibase-Mn [52]. These results support the previously suggested role of this histidine residue in catalysis through an orientation effect augmenting the probability of a near in-line attack of the metal-bridging water over the P-O scissile bond [52, 80]. The action of the GNH[D/E] histidine of ADPRibase-Mn as a general acid catalyst seems unlikely in view of the modest or null effect of the mutation on the kinetic parameters of the hydrolysis of the 2´,3´-cAMP phosphodiester, as opposed to the strong effect on the hydrolysis of the phosphoanhydride linkage of the other substrates (Table 1, and [52]). The importance of general acid catalysis should be, if something, more obvious for the hydrolysis of a phosphodiester than a phosphoanhydride, as the former would profit from a proton donor to yield an alcohol leaving group. A similar reasoning can be made from the results of the H111N mutation, as being the asparagine side chain unable to act as general catalyst, the results of this substitution did neither affect the kinetic parameters of 2´,3´-cAMP phosphodiester hydrolysis. On the other hand, concerning the possible orientation effect, the (near) independence of 2´,3´-cAMP hydrolysis on the GNH[D/E] histidine would be explainable by the structural rigidity of this substrate and its straightforward fit in the active site with the in-line orientation [52].

### The unique structural element s2s3 in human ADPRibase-Mn: catalytic role of Arg^43^ and role of Phe^37^ in the enzyme preference for ADP-ribose

In the human ADPRibase-Mn, the unique region s2s3 is formed by amino acids 30–45 (S5 Fig.). From the mutagenesis experiments, the two s2s3 amino acids that interacted with ADP-ribose, Arg^43^ and Phe^37^, emerged as critically relevant to the catalytic mechanism or the specificity.

The sidechain of Arg^43^ points towards the pyrophosphate groups of ADP-ribose (Fig. 2) and probably of the other docked substrates (S4 Fig.). This residue is invariant in the ADPRibase-Mn-like family (S3 Fig.), and it could have a role in stabilizing the transition state and or the leaving group. In agreement with this catalytic role, the R43A mutant showed a drastic, four-orders-of-magnitude decrease of catalytic efficiency (*k*_cat_/*K*_m_) with all the substrates except 2´,3´-cAMP, for which only a 200-fold decrease of efficiency was observed (Table 1). This, though quite strong an effect, indicates, together with the modest or null effects of His^111^ mutations, that the catalytic requirements for the hydrolysis of 2´,3´-cAMP by human ADPRibase-Mn are lesser than for the other substrates (see above: Table 1 and [52]).

In the hypothetical conformation adopted by ADP-ribose in the docking model, the hydrophobic ring of Phe^37^ made a contact with the nitrogenous base, which was not observed with the other docked substrates (S4 Fig.). Since the ADPRibase-Mn preference for ADP-ribose was characterized by a considerably lower *K*_m_ when compared to the other substrates (Table 1), the potential specificity of the interaction with Phe^37^ suggested that this amino acid could be a determinant for the ADP-ribose preference. In agreement with this view, the F37A mutant displayed a 19-fold increased *K*_m_ for ADP-ribose, with only a 2–3-fold increase of the CDP-choline and 2´,3´-cAMP *K*_m_ values. At the same time only minor decreases of *k*_cat_ were elicited by the same mutation. In terms of *k*_cat_/*K*_m_, the F37A mutation diminished the efficiency of ADP-ribose hydrolysis by 50 fold, but that of the other substrates, including cADPR, was reduced by only 3–4 fold. The control mutant F37Y showed the same kinetic parameters as the wild type. In summary, the preferred substrate of F37A-ADPRibase-Mn was CDP-choline, indicating that the preference of wild type ADPRibase-Mn by ADP-ribose is determined by Phe^37^. This residue is widely conserved as phenylalanine or tyrosine, in animals and plants with some substitutions by aliphatic amino acids in protist ADPRibase-Mn proteins (S3 Fig.). It would be interesting to learn the natural specificity of proteins of this family having Phe^37^ replaced by a nonaromatic residue.

Additional support for the interaction of ADP-ribose with Phe^37^ within s2s3 came from simulations of molecular dynamics (see below).

### Effects of docked ADP-ribose on the simulated molecular dynamics of the ADPRibase-Mn model

To search for possible effects of the binding of ADP-ribose to the active site of human ADPRibase-Mn, 40-ns simulations of molecular dynamics of the ADPRibase-Mn:ADP-ribose model obtained by docking, and of the free enzyme, were run and compared (Fig. 3). In the dynamic trajectory, four protein regions showed a substrate-dependent decrease of mobility, one of them apparently related to the proximity of bound ADP-ribose. The mobility of the backbone C atoms was quantitated by measuring their root mean square fluctuations (RMSF) along the trajectories, which identified the regions of diminished mobility as those formed by amino acids 7–14, 34–41, 78–84 and 333–342. The first and the last of those regions correspond to the polypeptide ends. Interestingly, region 34–41 is included in s2s3 (amino acids 30–45) and region 78–84 coincides well with h2 (amino acids 78–85), which have been identified as regions unique to the ADPRibase-Mn-like family (Fig. 1, S5 Fig. and [52]). On the other hand, region h7h8 (amino acids 170–212), also unique to this family, showed high mobility in its central part (amino acids 175–205) but was little affected by the presence of bound ADP-ribose in the simulation (Fig. 3).

In summary, the decreased mobility of the s2s3 element (which contains the ADP-ribose-specificity determinant Phe^37^) in the presence of docked ADP-ribose, supports the importance of the interaction of the substrate with the amino acid.

### The unique structural element h7h8 in human ADPRibase-Mn: relevance of Leu^196^ and Phe^210^

The unique structural element h7h8 of human ADPRibase-Mn is formed by amino acids 170–212 (S5 Fig.). Within it, we focused on Leu^196^ and Phe^210^. To judge from single-point mutations, only the latter had a clearcut repercussion over enzyme action. However, some relevance of Leu^196^ was inferred from the analysis of double mutants.

Leu^196^ is conserved in vertebrates but not in most of the ADPRibase-Mn proteins belonging to other taxa (S3 Fig.). This amino acid is located approximately opposite the specificity determinant Phe^37^, not very far from it across the entrance to the active site (Fig. 1). Together, these two amino acids could favor a closed conformation of the active site. In a docking model of ADP-ribose with rat ADPRibase-Mn, Leu^196^ is close to the adenine ring [2], but a similar contact was not seen in the docking complex with the modeled human enzyme (Fig. 2). However, mutagenesis of Leu^196^ had interesting if subtle effects. The L196A point mutation caused only a modest 2–5-fold decrease of catalytic efficiency with the four substrates tested. The F37A+L196A double mutant showed a modest enhancement of the inhibitory effect over that of the F37A point mutation. Unexpectedly, such effect was even slightly stronger with the control double mutation F37A+L196F, except for the activity on cADPR, which was less affected than by F37A+L196A (Table 1). The effect of Leu^196^ mutagenesis is best illustrated by the ratio of catalytic efficiencies ADP-ribose/cADPR: 150 for the wild type; 9.2 for F37A; 6.7 for F37A+L196A; 3.0 for F37A+L196F. The minor effect of the Leu^196^ substitutions, that favored the cADPR phosphohydrolase activity in relative terms, was taken to advantage, in a synergistic combination with the C253A mutation, to obtain an ADPRibase-Mn triple mutant that displayed preferential activity on cADPR (see below).

The sidechain of Phe^210^, which is widely conserved in the ADPRibase-Mn family (S3 Fig.), lays beside the substrate, near the pyrophosphate and the two riboses in the docking model with ADP-ribose, acting perhaps as a stanchion that keeps the substrate into position (Fig. 2). Its relevance was supported by the effects of the F210A mutation, which lowered 40–70 fold the catalytic efficiency for ADP-ribose, CDP-choline and 2´,3´-cAMP hydrolysis, and 500 fold for cADPR (Table 1). The decrease of *k*_cat_/*K*_m_ was due to increases of *K*_m_ for ADP-ribose and CDP-choline, with little effect on *k*_cat_ values, but mainly to a decrease of *k*_cat_ for 2´,3´-cAMP. The different response of the latter to the F210A mutation could be related to the rigidity of 2´,3´-cAMP and to a lower probability of adopting a conformation with the scissile P-O bond in line with the water nucleophile in the absence of the phenyl group of Phe^210^. The more flexible structures of ADP-ribose and CDP-choline, though binding with lower affinities in the absence of the aromatic ring of Phe^210^, would then be as likely to adopt the conformation favorable to the nucleophilic attack as when bound to the wild-type enzyme.

### Role of Cys^253^ as a factor limiting the cADPR phosphohydrolase activity of ADPRibase-Mn

Most of the mutations tested either diminished the catalytic efficiency of cADPR phosphohydrolase or elicited only modest changes, more or less in parallel to changes of efficiency on the other substrates considered (Table 1). Anyhow, there were two important exceptions to this general trend. One was observed in the effects of the F37A mutation which, as discussed below, revealed Phe^37^ is a major determinant of the preference of ADPRibase-Mn for ADP-ribose as substrate, with a comparatively minor effect on the hydrolysis of the other substrates, cADPR included. The other exception, more relevant to the character of the cADPR phosphohydrolase activity, was the effect of the C253A mutation, which unexpectedly elicited a specific increase (10 fold) of the catalytic efficiency over cADPR, composed by a decrease of the *K*_m_ (to a value similar to those of the rat and the zebrafish enzymes), and an increase of *k*_cat_. In comparison, the removal of the thiol group of Cys^253^ did not affect or caused a very slight increase of the apparent affinity of the enzyme for the other substrates. It seems that this residue hinders the optimal binding of cADPR to the active site of human ADPRibase-Mn.

Cys^253^ is extensively conserved among vertebrates, but not in ADPRibase-Mn-like proteins of other taxa in which the family is present (S3 Fig.). Whether non-vertebrate enzymes, particularly those of higher plant origin in which alanine occupies naturally the position equivalent to human Cys^253^, could be better cADPR phosphohydrolase catalysts than mammalian ADPRibase-Mn remains speculative.

### Construction of a triple mutant preferentially active on cADPR

As a proof of principle that the specificity for cADPR can be augmented by mutagenesis, the triple ADPRibase-Mn mutant F37A+L196F+C253A was constructed. In it, the double mutation F37A+L196F, which had been shown to lessen the relative preference for ADP-ribose *versus* cADPR, was combined with the specific activation of cADPR phosphohydrolase produced by the C253A mutation in absolute terms. Had this combination produced additive effects, one would expect that the resulting protein displayed a ratio of catalytic efficiencies cADPR/ADP-ribose about 2. In fact, the kinetic characterization of the triple mutant gave a *k*_cat_/*K*_m_ value of 35500 M^-1^s^-1^, 3.2-fold higher than the efficiency of ADP-ribose hydrolysis, and also higher than those of CDP-choline and 2´,3´-cAMP (Table 1). The *k*_cat_/*K*_m_ values for other ADPRibase-Mn substrates not included in the kinetic study of mutants were also assayed with the triple mutant to proof that they were also lower than with cADPR, namely CDP-glycerol, 22000 ± 4000 M^-1^s^-1^; CDP-ethanolamine, 5800 ± 700 M^-1^s^-1^; ADP, 53 ± 12 M^-1^s^-1^.

### Concluding discussion

First, we would like to emphasize that the structural and dynamic information on human ADPRibase-Mn presented in this study comes from homology modeling and simulation studies of docking and molecular dynamics. The strength of this approach is not comparable to what NMR or X-ray studies of actual structures and interactions would afford. In particular, one has to be aware that the docking itself produces speculative information and may give false positives. Therefore, data relative to interactions, positions, angles or distances measured on the human ADPRibase-Mn models should be taken as hypothetical. We have used these data as starting point to choose residues for mutation, and as arguments to help in the interpretation of the kinetic behavior of mutant proteins.

The dinuclear center of the crystal structure of zebrafish ADPRibase-Mn contains a metal-bridging molecule of water that was included in the homology model of the human enzyme to which the substrates were docked. Based mainly on the docking of ADP-ribose to this model, eight amino acids of human ADPRibase-Mn were selected for mutagenesis. To analyze the results of the mutagenesis and enzyme kinetics studies, it was hypothesized that the bridging metal-activated water or hydroxide could be the attacking nucleophile like in the mechanisms of other phosphoesterases [61, 69]. According to this model, substrates need correct positioning in the active center for the attack to be made under near in-line conditions. Another mechanistic aspect here considered is the possible stabilization of the transition state of the reactions by charge neutralization.

Several ADPRibase-Mn amino acids with roles in catalysis and/or substrate specificity have been identified in this study. In many cases, the kinetic effects of a particular mutation were different depending on the substrate considered. A corollary of this picture is that the correct positioning of each substrate may depend, more or less strictly, on a different set of amino acids. Not surprisingly, substrate-positioning residues may have bearing both on catalysis (*k*_cat_) and substrate binding (*K*_m_).

Two amino acids appeared clearly involved in catalysis. (i) Arg^43^, due to the proximity of its sidechain end to the substrate phosphoryl group(s), probably stabilizes the transition state and/or the leaving group by charge neutralization. Comparing the hydrolytic reactions with different substrates, the bearing of Arg^43^ on catalysis was less dramatic (but quite strong at any rate) for the hydrolysis of 2´,3´-cAMP, perhaps because of the single phosphoryl group, and the relatively low activation energy of hydrolysis of its P-O2´ ester linkage [82]. (ii) His^111^, that may form a hydrogen bond with the O atom of the scissile P-O bond, probably helps to keep the substrate in a position susceptible of in-line nucleophilic attack by the metal-activated water. Again, the bearing of His^111^ on catalysis was less marked with 2´,3´-cAMP as the substrate. As a consequence, this was the best substrate for the H111A-ADPRibase-Mn mutant. A similar effect of the equivalent residue and mutation, observed with zebrafish ADPRibase-Mn, has been attributed to the low conformational flexibility of this compound and to its lesser need of assisted orientation towards the in-line position [52]. Another substrate-specific effect of His^111^ concerned substrate binding, since its mutation increased the *K*_m_ for CDP-choline, but not for the other substrates.

Another two residues appeared clearly involved in substrate specificity. (i) Phe^37^ is located in the rim of the entrance to the active site, distant from the catalytic core around the dinuclear center. In such position, Phe^37^ acts as the determinant of the ADPRibase-Mn preference for ADP-ribose, possibly due to a specific interaction of the aromatic residue with the nitrogenous base of ADP-ribose. The other substrates do not seem to make this contact in their docking models. The larger observed effect of the F37A mutation was the increase of the *K*_m_ for ADP-ribose, with only modest effects on the *k*_cat_ values or on the *K*_m_ s for CDP-choline and 2´,3´-cAMP. Therefore, the best substrate of the F37A mutant was no longer ADP-ribose, but CDP-choline. (ii) Cys^253^ is located near the catalytic core and the dinuclear center, without evidence for interaction with the metals. The SH group is at near contact distance with the C3 of the non-nucleosidic ribose of ADP-ribose or the “northern” ribose of cADPR. Interestingly, this does not seem to be a favorable contact, but a subtle obstacle to optimal cADPR binding and positioning, thus limiting the phosphohydrolytic activity on this substrate. The C253A mutation elicited modest but favorable effects both in *k*_cat_ and *K*_m_ for cADPR, what brought about a significant and unique increase in catalytic efficiency on this substrate. This was the only point mutation bringing about an efficiency increase. Two other mutations together (F37A and L196A), although eliciting negative effects in absolute terms, affected less the hydrolysis of cADPR than of other substrates. The result of combining the three mutations (F37A+L196F+C253A) was an ADPRibase-Mn protein that acted preferentially as cADPR phosphohydrolase.

In summary, this work sheds light on the mechanism and specificity of human ADPRibase-Mn and paves the way for identifying more specific cADPR phosphohydrolases, perhaps among ADPRibase-Mn-like proteins in which Cys^253^ is not conserved, and/or for designing ADPRibase-Mn mutants with such specificity. This future goal could be compared to the reported conversion of CD38 by mutagenesis from a predominant NAD glycohydrolase to a specific ADP-ribosyl cyclase [83]. A specific cADPR phosphohydrolase would make a useful tool for in vitro analytical and in vivo functional studies of this regulator.

## Supporting Information

S1 Dataset Coordinates of the docking complex of the model structure of human ADPRibase-Mn with ADP-ribose. (PDB)

S2 Dataset Coordinates of the docking complex of the model structure of human ADPRibase-Mn with cADPR. (PDB)

S3 Dataset Coordinates of the docking complex of the model structure of human ADPRibase-Mn with CDP-choline. (PDB)

S4 Dataset Coordinates of the docking complex of the model structure of human ADPRibase-Mn with 2´,3´-cAMP. (PDB)

S1 Fig pH requirements of human ADPRibase-Mn activities. The assays were performed with 500 μM substrate in the presence of 5 mM MnCl_2_ at the indicated pH values using 100 mM Tris/acetate (pH 6.03 and 6.67), 100 mM Tris/HCl (pH 6.55, 7.08, 7.46, 7.73, 8.29, 8.55, 8.94 and 9.68) or 100 mM glycine/NaOH (8.89, 9.93 and 10.50). The pH values were measured with a glass electrode in reaction mixtures at the assay temperature of 37°C. The results are means with S.D. of three experiments.
(PDF)

S2 Fig Mn^2+^ requirements of human ADPRibase-Mn activities. In this experiment the means ± S.D. were obtained in triplicate assays performed at pH 7.5, with 500 μM ADP-ribose or CDP-choline, or with 2500 μM 2´,3´-cAMP, in the presence of the indicated MnCl_2_ concentrations. The response of activity to Mn^2+^ concentration (5 μM-5000 μM) is representative of several other experiments performed under different experimental conditions.
(PDF)

S3 Fig Sequence alignment of 108 eukaryotic proteins representative of the ADPRibase-Mn-like family. The names of species and GenBank accession numbers of the proteins are colored according to the following code: *black*, vertebrates; *blue*, invertebrate animals; *red*, plants; *orange*, protists. All the proteins were sorted out from a BlastP search [57] run on July 7, 2014. The proteins shown were selected applying criteria described under Materials and Methods, except for the selection of a few additional proteins: (i) the one from *Mus musculus*, despite being 96% identical to that from *Rattus norvegicus*, and due to its classification as the product of an immune gene [2, 37]; (ii) those from *Bos taurus* and *Ovis aries*, despite being 97% identical, because these species are included in NCBI RefSeq Genomes; (iii) those from the bivalve *Crassostrea gigas*, the snail *Lottia gigantea*, and the worm *Helobdella robusta*, despite not being yet recorded in NCBI_RefSeq, due to their importance as possible representatives of the protein family in invertebrate animal phyla. Residues identically conserved in all sequences are denoted by white letters on red background. Other regions of high sequence conservation are indicated by boxed red letters on white background. The numbers above the sequences correspond to the human protein. *Blue triangles* mark the amino acids that were mutated in this study. *Red rectangles* mark the five short regions that together form the disperse amino acid motif of the MDP superfamily. The unique regions s2s3, h2 and h7h8, typical of the ADPRibase-Mn family [52], are also indicated above the human protein.
(PDF)

S4 Fig Stereograms of substrates docked to the active site of the model structure of human ADPRibase-Mn. The views show metals, metal-bridging water, and amino acids identified by their interaction with docked ADP-ribose and tested by mutagenesis (see the main text for details). The models illustrate the hypothesis that the metal bridging water, according to its location, could be the attacking nucleophile in the ADPRibase-Mn reactions. The nucleophilic attack distances are drawn only to emphasize this hypothesis.
(PDF)

S5 Fig Topology diagram of the model structure of human ADPRibase-Mn. Structural elements (triangle, strand; circles, helix) are shown according to their numbering (strands 1–15 and helices 1–12) in zebrafish ADPRibase-Mn [52]. All the other numbers identify residues of human ADPRibase-Mn. The small golden circles are the metals of the dinuclear center; the eight amino acids of human ADPRibase-Mn coordinated to the metals are indicated. Strand 6 and helix 8 are present in the zebrafish protein; however, in the human protein, strand 6 is just a beta bridge and helix 8 was missing in the model downloaded from SWISS-MODEL to prepare the complete model structure of human ADPRibase-Mn (see Materials and Methods and Fig. 1), but appears in more recent models in the same repository [53].
(PDF)

S1 Table Forward primers used for site-directed mutagenesis. The reverse primers were the reverse complements of those shown.
(PDF)

S2 Table Purity of the recombinant protein preparations. In each case, enzyme purity was estimated by Coomassie blue-stained SDS-PAGE followed by image analysis quantitation.
(PDF)

S3 Table Substrate specificity of human ADPRibase-Mn. The activities were assayed in triplicate with 500 μM substrate in the presence of 5 mM MnCl_2_. The results are mean values with S.D. The sensitivity limit of the assay is indicated when no activity was detected.
(PDF)

## References

[1] Canales J, Pinto RM, Costas MJ, Hernández MT, Miró A, Bernet D, et al Rat liver nucleoside diphosphosugar or diphosphoalcohol pyrophosphatases different from nucleotide pyrophosphatase or phosphodiesterase I: substrate specificities of Mg^2+^- and/or Mn^2+^-dependent hydrolases acting on ADP-ribose. Biochim Biophys Acta. 1995;1246: 167–177. 7819284 10.1016/0167-4838(94)00191-i

[2] Canales J, Fernández A, Ribeiro JM, Cabezas A, Rodrigues JR, Cameselle JC, et al Mn^2+^-dependent ADP-ribose/CDP-alcohol pyrophosphatase: a novel metallophosphoesterase family preferentially expressed in rodent immune cells. Biochem J. 2008;413: 103–113. 10.1042/BJ20071471 18352857

[3] Ash DE, Schramm VL. Determination of free and bound manganese(II) in hepatocytes from fed and fasted rats. J Biol Chem. 1982;257: 9261–9264. 6286611

[4] Canales J, Fernández A, Rodrigues JR, Ferreira R, Ribeiro JM, Cabezas A, et al Hydrolysis of the phosphoanhydride linkage of cyclic ADP-ribose by the Mn^2+^-dependent ADP-ribose/CDP-alcohol pyrophosphatase. FEBS Lett. 2009;583: 1593–1598. 10.1016/j.febslet.2009.04.023 19379742

[5] Kuhn FJ, Luckhoff A. Sites of the NUDT9-H domain critical for ADP-ribose activation of the cation channel TRPM2. J Biol Chem. 2004;279: 46431–46437. 15347676 10.1074/jbc.M407263200

[6] Kolisek M, Beck A, Fleig A, Penner R. Cyclic ADP-ribose and hydrogen peroxide synergize with ADP-ribose in the activation of TRPM2 channels. Mol Cell. 2005;18: 61–69. 15808509 10.1016/j.molcel.2005.02.033

[7] Perraud AL, Takanishi CL, Shen B, Kang S, Smith MK, Schmitz C, et al Accumulation of free ADP-ribose from mitochondria mediates oxidative stress-induced gating of TRPM2 cation channels. J Biol Chem. 2005;280: 6138–6148. 15561722 10.1074/jbc.M411446200

[8] Gasser A, Glassmeier G, Fliegert R, Langhorst MF, Meinke S, Hein D, et al Activation of T cell calcium influx by the second messenger ADP-ribose. J Biol Chem. 2006;281: 2489–2496. 16316998 10.1074/jbc.M506525200

[9] Yu P, Wang Q, Zhang LH, Lee HC, Zhang L, Yue J. A cell permeable NPE caged ADP-ribose for studying TRPM2. PLoS One. 2012;7: e51028 10.1371/journal.pone.0051028 23236422 PMC3517590

[10] Clementi E, Riccio M, Sciorati C, Nistico G, Meldolesi J. The type 2 ryanodine receptor of neurosecretory PC12 cells is activated by cyclic ADP-ribose. Role of the nitric oxide/cGMP pathway. J Biol Chem. 1996;271: 17739–17745. 8663443 10.1074/jbc.271.30.17739

[11] Ogunbayo OA, Zhu Y, Rossi D, Sorrentino V, Ma J, Zhu MX, et al Cyclic adenosine diphosphate ribose activates ryanodine receptors, whereas NAADP activates two-pore domain channels. J Biol Chem. 2011;286: 9136–9140. 10.1074/jbc.M110.202002 21216967 PMC3058984

[12] Lee HC. Cyclic ADP-ribose and nicotinic acid adenine dinucleotide phosphate (NAADP) as messengers for calcium mobilization. J Biol Chem. 2012;287: 31633–31640. 10.1074/jbc.R112.349464 22822066 PMC3442497

[13] Ferrero E, Lo Buono N, Horenstein AL, Funaro A, Malavasi F. The ADP-ribosyl cyclases—the current evolutionary state of the ARCs. Front Biosci. 2014;19: 986–1002. 24896331 10.2741/4262

[14] Lee HC, Aarhus R. ADP-ribosyl cyclase: an enzyme that cyclizes NAD^+^ into a calcium-mobilizing metabolite. Cell Regul. 1991;2: 203–209. 1830494 10.1091/mbc.2.3.203 PMC361752

[15] Inageda K, Takahashi K, Tokita K, Nishina H, Kanaho Y, Kukimoto I, et al Enzyme properties of *Aplysia* ADP-ribosyl cyclase: comparison with NAD glycohydrolase of CD38 antigen. J Biochem. 1995;117: 125–131. 7775378 10.1093/oxfordjournals.jbchem.a124698

[16] Liu Q, Graeff R, Kriksunov IA, Jiang H, Zhang B, Oppenheimer N, et al Structural basis for enzymatic evolution from a dedicated ADP-ribosyl cyclase to a multifunctional NAD hydrolase. J Biol Chem. 2009;284: 27637–27645. 10.1074/jbc.M109.031005 19640846 PMC2785692

[17] Takasawa S, Tohgo A, Noguchi N, Koguma T, Nata K, Sugimoto T, et al Synthesis and hydrolysis of cyclic ADP-ribose by human leukocyte antigen CD38 and inhibition of the hydrolysis by ATP. J Biol Chem. 1993;268: 26052–26054. 8253715

[18] Kim H, Jacobson EL, Jacobson MK. Synthesis and degradation of cyclic ADP-ribose by NAD glycohydrolases. Science. 1993;261: 1330–1333. 8395705 10.1126/science.8395705

[19] Howard M, Grimaldi JC, Bazan JF, Lund FE, Santos-Argumedo L, Parkhouse RM, et al Formation and hydrolysis of cyclic ADP-ribose catalyzed by lymphocyte antigen CD38. Science. 1993;262: 1056–1059. 8235624 10.1126/science.8235624

[20] Zocchi E, Franco L, Guida L, Benatti U, Bargellesi A, Malavasi F, et al A single protein immunologically identified as CD38 displays NAD^+^ glycohydrolase, ADP-ribosyl cyclase and cyclic ADP-ribose hydrolase activities at the outer surface of human erythrocytes. Biochem Biophys Res Commun. 1993;196: 1459–1465. 8250903 10.1006/bbrc.1993.2416

[21] Hirata Y, Kimura N, Sato K, Ohsugi Y, Takasawa S, Okamoto H, et al ADP ribosyl cyclase activity of a novel bone marrow stromal cell surface molecule, BST-1. FEBS Lett. 1994;356: 244–248. 7805847 10.1016/0014-5793(94)01279-2

[22] Berthelier V, Tixier JM, Muller-Steffner H, Schuber F, Deterre P. Human CD38 is an authentic NAD(P)^+^ glycohydrolase. Biochem J. 1998;330: 1383–1390. 9494110 10.1042/bj3301383 PMC1219286

[23] Liu Q, Kriksunov IA, Graeff R, Lee HC, Hao Q. Structural basis for formation and hydrolysis of the calcium messenger cyclic ADP-ribose by human CD38. J Biol Chem. 2007;282: 5853–5861. 17182614 10.1074/jbc.M609093200

[24] Egea PF, Muller-Steffner H, Kuhn I, Cakir-Kiefer C, Oppenheimer NJ, Stroud RM, et al Insights into the mechanism of bovine CD38/NAD^+^glycohydrolase from the X-ray structures of its Michaelis complex and covalently-trapped intermediates. PLoS One. 2012;7: e34918 10.1371/journal.pone.0034918 22529956 PMC3329556

[25] Gu Q-M, Sih CJ. Cyclic ADP-ribose: synthesis and structural assignment. J Am Chem Soc. 1994;116: 7481–7486.

[26] Khorana HG. Phosphodiesterases In: Boyer PD, Lardy HA, Myrbäck K, editors. The Enzymes, 2nd ed, Vol 5 New York: Academic Press; 1961 p. 79–94.

[27] Razzell WE. Phosphodiesterases. Methods Enzymol. 1963;6: 236–258.

[28] García-Díaz M, Ávalos M, Cameselle JC. Alcohol esterification reactions and mechanisms of snake venom 5′-nucleotide phosphodiesterase. Eur J Biochem. 1993;213: 1139–1148. 8389294 10.1111/j.1432-1033.1993.tb17864.x

[29] Sana TR, Gordon DB, Fischer SM, Tichy SE, Kitagawa N, Lai C, et al Global mass spectrometry based metabolomics profiling of erythrocytes infected with *Plasmodium falciparum* . PLoS One. 2013;8: e60840 10.1371/journal.pone.0060840 23593322 PMC3621881

[30] Guse AH, Cakir-Kiefer C, Fukuoka M, Shuto S, Weber K, Bailey VC, et al Novel hydrolysis-resistant analogues of cyclic ADP-ribose: modification of the “northern” ribose and calcium release activity. Biochemistry. 2002;41: 6744–6751. 12022878 10.1021/bi020171b

[31] Guse AH. Biochemistry, biology, and pharmacology of cyclic adenosine diphosphoribose (cADPR). Curr Med Chem. 2004;11: 847–855. 15078169 10.2174/0929867043455602

[32] Shuto S, Matsuda A. Chemistry of cyclic ADP-ribose and its analogs. Curr Med Chem. 2004;11: 827–845. 15078168 10.2174/0929867043455639

[33] Moreau C, Liu Q, Graeff R, Wagner GK, Thomas MP, Swarbrick JM, et al CD38 structure-based inhibitor design using the 1-cyclic inosine 5′-diphosphate ribose template. PLoS One. 2013;8: e66247 23840430 10.1371/journal.pone.0066247 PMC3686795

[34] Huang X, Dong M, Liu J, Zhang K, Yang Z, Zhang L, et al Concise syntheses of trifluoromethylated cyclic and acyclic analogues of cADPR. Molecules. 2010;15: 8689–8701. 10.3390/molecules15128689 21119564 PMC6259357

[35] Wu H, Yang Z, Zhang L, Zhang L. Concise synthesis of novel acyclic analogues of cADPR with an ether chain as the northern moiety. New J Chem. 2010;34: 956–966.

[36] Swarbrick JM, Graeff R, Garnham C, Thomas MP, Galione A, Potter BV. ‘Click cyclic ADP-ribose’: a neutral second messenger mimic. Chem Commun (Camb). 2014;50: 2458–2461. 10.1039/c3cc49249d 24452494 PMC4047616

[37] Hutton JJ, Jegga AG, Kong S, Gupta A, Ebert C, Williams S, et al Microarray and comparative genomics-based identification of genes and gene regulatory regions of the mouse immune system. BMC Genomics. 2004;5: 82 15504237 10.1186/1471-2164-5-82 PMC534115

[38] Malavasi F, Deaglio S, Ferrero E, Funaro A, Sancho J, Ausiello CM, et al CD38 and CD157 as receptors of the immune system: a bridge between innate and adaptive immunity. Mol Med. 2006;12: 334–341. 17380201 10.2119/2006-00094.Malavasi PMC1829205

[39] Partida-Sánchez S, Rivero-Nava L, Shi G, Lund FE. CD38: an ecto-enzyme at the crossroads of innate and adaptive immune responses. Adv Exp Med Biol. 2007;590: 171–183. 17191385 10.1007/978-0-387-34814-8_12

[40] Postigo J, Iglesias M, Cerezo-Wallis D, Rosal-Vela A, Garcia-Rodriguez S, Zubiaur M, et al Mice deficient in CD38 develop an attenuated form of collagen type II-induced arthritis. PLoS One. 2012;7: e33534 10.1371/journal.pone.0033534 22438945 PMC3306406

[41] Ernst IM, Fliegert R, Guse AH. Adenine dinucleotide second messengers and T-lymphocyte calcium signaling. Front Immunol. 2013;4: 259 10.3389/fimmu.2013.00259 24009611 PMC3756424

[42] De Flora A, Guida L, Franco L, Zocchi E. The CD38/cyclic ADP-ribose system: a topological paradox. Int J Biochem Cell Biol. 1997;29: 1149–1166. 9438379 10.1016/s1357-2725(97)00062-9

[43] Shrimp JH, Hu J, Dong M, Wang BS, MacDonald R, Jiang H, et al Revealing CD38 cellular localization using a cell permeable, mechanism-based fluorescent small-molecule probe. J Am Chem Soc. 2014;136: 5656–5663. 10.1021/ja411046j 24660829 PMC4004212

[44] Song EK, Rah SY, Lee YR, Yoo CH, Kim YR, Yeom JH, et al Connexin-43 hemichannels mediate cyclic ADP-ribose generation and its Ca^2+^-mobilizing activity by NAD^+^/cyclic ADP-ribose transport. J Biol Chem. 2011;286: 44480–44490. 10.1074/jbc.M111.307645 22033928 PMC3247979

[45] Xu M, Li XX, Ritter JK, Abais JM, Zhang Y, Li PL. Contribution of NADPH oxidase to membrane CD38 internalization and activation in coronary arterial myocytes. PLoS One. 2013;8: e71212 10.1371/journal.pone.0071212 23940720 PMC3737089

[46] Ribeiro JM, Carloto A, Costas MJ, Cameselle JC. Human placenta hydrolases active on free ADP-ribose: an ADP-sugar pyrophosphatase and a specific ADP-ribose pyrophosphatase. Biochim Biophys Acta. 2001;1526: 86–94. 11287126 10.1016/s0304-4165(01)00113-1

[47] Perraud AL, Shen B, Dunn CA, Rippe K, Smith MK, Bessman MJ, et al NUDT9, a member of the Nudix hydrolase family, is an evolutionarily conserved mitochondrial ADP-ribose pyrophosphatase. J Biol Chem. 2003;278: 1794–1801. 12427752 10.1074/jbc.M205601200

[48] Carloto A, Costas MJ, Cameselle JC, McLennan AG, Ribeiro JM. The specific, submicromolar-*K* _m_ ADP-ribose pyrophosphatase purified from human placenta is enzymically indistinguishable from recombinant NUDT9 protein, including a selectivity for Mn^2+^ as activating cation and increase in *K* _m_ for ADP-ribose, both elicited by H_2_O_2_ . Biochim Biophys Acta. 2006;1760: 1545–1551. 16860484 10.1016/j.bbagen.2006.06.003

[49] Zha M, Zhong C, Peng Y, Hu H, Ding J. Crystal structures of human NUDT5 reveal insights into the structural basis of the substrate specificity. J Mol Biol. 2006;364: 1021–1033. 17052728 10.1016/j.jmb.2006.09.078

[50] Andreeva A, Howorth D, Chothia C, Kulesha E, Murzin AG. SCOP2 prototype: a new approach to protein structure mining. Nucleic Acids Res. 2014;42: D310–314. 10.1093/nar/gkt1242 24293656 PMC3964979

[51] Sillitoe I, Cuff AL, Dessailly BH, Dawson NL, Furnham N, Lee D, et al New functional families (FunFams) in CATH to improve the mapping of conserved functional sites to 3D structures. Nucleic Acids Res. 2013;41: D490–498. 10.1093/nar/gks1211 23203873 PMC3531114

[52] Rodrigues JR, Fernández A, Canales J, Cabezas A, Ribeiro JM, Costas MJ, et al Characterization of *Danio rerio* Mn^2+^-dependent ADP-ribose/CDP-alcohol diphosphatase, the structural prototype of the ADPRibase-Mn-like protein family. PLoS One. 2012;7: e42249 10.1371/journal.pone.0042249 22848751 PMC3407115

[53] Kiefer F, Arnold K, Kunzli M, Bordoli L, Schwede T. The SWISS-MODEL Repository and associated resources. Nucleic Acids Res. 2009;37: D387–392. 10.1093/nar/gkn750 18931379 PMC2686475

[54] Markley JL, Aceti DJ, Bingman CA, Fox BG, Frederick RO, Makino S, et al The Center for Eukaryotic Structural Genomics. J Struct Funct Genomics. 2009;10: 165–179. 10.1007/s10969-008-9057-4 19130299 PMC2705709

[55] Bradford MM. A rapid and sensitive method for the quantitation of microgram quantities of protein utilizing the principle of protein-dye binding. Anal Biochem. 1976;72: 248–254. 942051 10.1016/0003-2697(76)90527-3

[56] Fehrst A. Structure and Mechanism in Protein Science A Guide to Enzyme Catalysis and Protein Folding. New York: W. Freeman & Co; 1998.

[57] Altschul SF, Wootton JC, Gertz EM, Agarwala R, Morgulis A, Schaffer AA, et al Protein database searches using compositionally adjusted substitution matrices. FEBS J. 2005;272: 5101–5109. 16218944 10.1111/j.1742-4658.2005.04945.x PMC1343503

[58] Pruitt KD, Tatusova T, Brown GR, Maglott DR. NCBI Reference Sequences (RefSeq): current status, new features and genome annotation policy. Nucleic Acids Res. 2012;40: D130–135. 10.1093/nar/gkr1079 22121212 PMC3245008

[59] Klabunde T, Sträter N, Fröhlich R, Witzel H, Krebs B. Mechanism of Fe(III)-Zn(II) purple acid phosphatase based on crystal structures. J Mol Biol. 1996;259: 737–748. 8683579 10.1006/jmbi.1996.0354

[60] Richter W. 3′,5′ Cyclic nucleotide phosphodiesterases class III: members, structure, and catalytic mechanism. Proteins. 2002;46: 278–286. 11835503 10.1002/prot.10049

[61] Mitic N, Smith SJ, Neves A, Guddat LW, Gahan LR, Schenk G. The catalytic mechanisms of binuclear metallohydrolases. Chem Rev. 2006;106: 3338–3363. 16895331 10.1021/cr050318f

[62] Shenoy AR, Capuder M, Draskovic P, Lamba D, Visweswariah SS, Podobnik M. Structural and biochemical analysis of the Rv0805 cyclic nucleotide phosphodiesterase from *Mycobacterium tuberculosis* . J Mol Biol. 2007;365: 211–225. 17059828 10.1016/j.jmb.2006.10.005

[63] Kim YG, Jeong JH, Ha NC, Kim KJ. Structural and functional analysis of the Lmo2642 cyclic nucleotide phosphodiesterase from *Listeria monocytogenes* . Proteins. 2011;79: 1205–1214. 10.1002/prot.22954 21246635

[64] Sievers F, Wilm A, Dineen D, Gibson TJ, Karplus K, Li W, et al Fast, scalable generation of high-quality protein multiple sequence alignments using Clustal Omega. Mol Syst Biol. 2011;7: 539 10.1038/msb.2011.75 21988835 PMC3261699

[65] Robert X, Gouet P. Deciphering key features in protein structures with the new ENDscript server. Nucleic Acids Res. 2014;42: W320–324. 10.1093/nar/gku316 24753421 PMC4086106

[66] Sali A, Blundell TL. Comparative protein modelling by satisfaction of spatial restraints. J Mol Biol. 1993;234: 779–815. 8254673 10.1006/jmbi.1993.1626

[67] Sanner MF. A component-based software environment for visualizing large macromolecular assemblies. Structure. 2005;13: 447–462. 15766546 10.1016/j.str.2005.01.010

[68] Morris GM, Goodsell DS, Halliday RS, Huey R, Hart WE, Belew RK, et al Automated docking using a Lamarckian genetic algorithm and an empirical binding free energy function. J Comput Chem. 1998;19: 1639–1662.

[69] Swingle MR, Honkanen RE, Ciszak EM. Structural basis for the catalytic activity of human serine/threonine protein phosphatase-5. J Biol Chem. 2004;279: 33992–33999. 15155720 10.1074/jbc.M402855200

[70] Hess B, Kutzner C, van der Spoel D, Lindahl E. GROMACS 4: Algorithms for highly efficient, load-balanced, and scalable molecular simulation. J Chem Theory Comput. 2008;4: 435–447. 26620784 10.1021/ct700301q

[71] Oostenbrink C, Villa A, Mark AE, van Gunsteren WF. A biomolecular force field based on the free enthalpy of hydration and solvation: the GROMOS force-field parameter sets 53A5 and 53A6. J Comput Chem. 2004;25: 1656–1676. 15264259 10.1002/jcc.20090

[72] Berendsen HJC, Postma JPM, Van Gunsteren WF, Hermans J. Interaction models for water in relation to protein hydration In: Pullman B, editor. Intermolecular Forces. Dordrecht: Reidel; 1981 p. 331–342.

[73] Hess B. P-LINCS: A parallel linear constraint solver for molecular simulation. J Chem Theory Comput. 2008;4: 116–122. 26619985 10.1021/ct700200b

[74] Hess B, Bekker H, Berendsen HJC, Fraaije JGEM. LINCS: A linear constraint solver for molecular simulations. J Comput Chem. 1997;18: 1463–1472.

[75] Miyamoto S, Kollman PA. SETTLE: An analytical version of the SHAKE and RATTLE algorithm for rigid water models. J Comput Chem. 1992;13: 952–962.

[76] Berendsen HJC, Postma JPM, Van Gunsteren WF, DiNola A, Haak JR. Molecular-dynamics with coupling to an external bath. J Chem Phys. 1984;81: 3684–3690.

[77] Kukimoto I, Hoshino S, Kontani K, Inageda K, Nishina H, Takahashi K, et al Stimulation of ADP-ribosyl cyclase activity of the cell surface antigen CD38 by zinc ions resulting from inhibition of its NAD^+^ glycohydrolase activity. Eur J Biochem. 1996;239: 177–182. 8706705 10.1111/j.1432-1033.1996.0177u.x

[78] Zhuo S, Clemens JC, Stone RL, Dixon JE. Mutational analysis of a Ser/Thr phosphatase. Identification of residues important in phosphoesterase substrate binding and catalysis. J Biol Chem. 1994;269: 26234–26238. 7929339

[79] Mertz P, Yu L, Sikkink R, Rusnak F. Kinetic and spectroscopic analyses of mutants of a conserved histidine in the metallophosphatases calcineurin and lambda protein phosphatase. J Biol Chem. 1997;272: 21296–21302. 9261141 10.1074/jbc.272.34.21296

[80] Funhoff EG, Wang Y, Andersson G, Averill BA. Substrate positioning by His92 is important in catalysis by purple acid phosphatase. FEBS J. 2005;272: 2968–2977. 15955057 10.1111/j.1742-4658.2005.04686.x

[81] Keppetipola N, Shuman S. A phosphate-binding histidine of binuclear metallophosphodiesterase enzymes is a determinant of 2′,3′-cyclic nucleotide phosphodiesterase activity. J Biol Chem. 2008;283: 30942–30949. 10.1074/jbc.M805064200 18757371 PMC2576524

[82] Rao F, Qi Y, Murugan E, Pasunooti S, Ji Q. 2′,3′-cAMP hydrolysis by metal-dependent phosphodiesterases containing DHH, EAL, and HD domains is non-specific: Implications for PDE screening. Biochem Biophys Res Commun. 2010;398: 500–505. 10.1016/j.bbrc.2010.06.107 20599695

[83] Graeff R, Liu Q, Kriksunov IA, Kotaka M, Oppenheimer N, Hao Q, et al Mechanism of cyclizing NAD to cyclic ADP-ribose by ADP-ribosyl cyclase and CD38. J Biol Chem. 2009;284: 27629–27636. 10.1074/jbc.M109.030965 19640843 PMC2785691

