# Supplementary figures and images for: Molecular Bases of Catalysis and ADP-Ribose Preference of Human Mn2+-Dependent ADP-Ribose/CDP-Alcohol Diphosphatase and Conversion by Mutagenesis to a Preferential Cyclic ADP-Ribose Phosphohydrolase
Source: PLoS One. 2015 Feb 18;10(2):e0118680. doi: 10.1371/journal.pone.0118680 (PMC4334965; doi:10.1371/journal.pone.0118680)

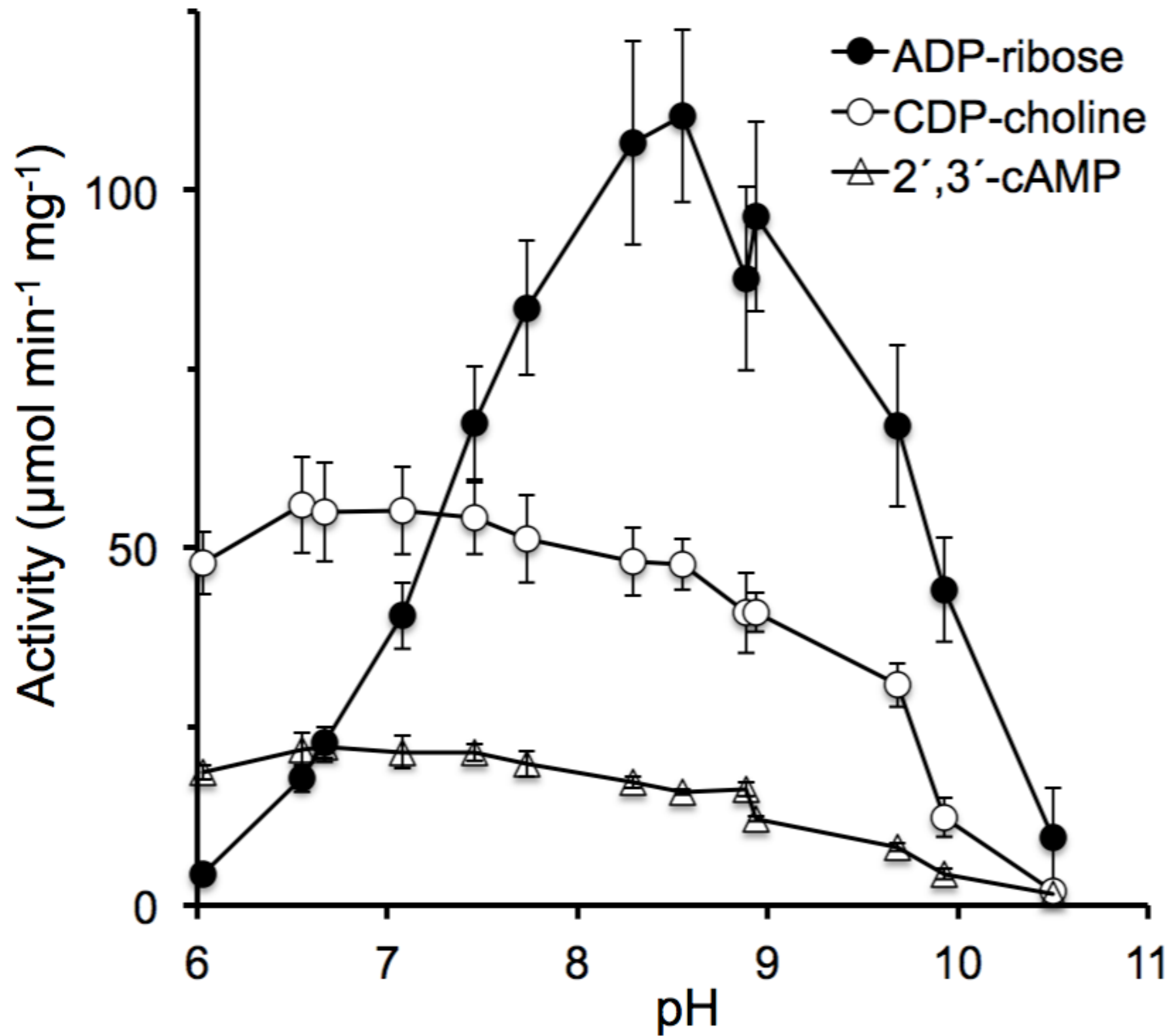

Supplement: S1 Fig — The assays were performed with 500 μM substrate in the presence of 5 mM MnCl2 at the indicated pH values using 100 mM Tris/acetate (pH 6.03 and 6.67), 100 mM Tris/HCl (pH 6.55, 7.08, 7.46, 7.73, 8.29, 8.55, 8.94 and 9.68) or 100 mM glycine/NaOH (8.89, 9.93 and 10.50). The pH values were measured with a glass electrode in reaction mixtures at the assay temperature of 37°C. The results are means with S.D. of three experiments. (PDF) [file pone.0118680.s005.pdf]

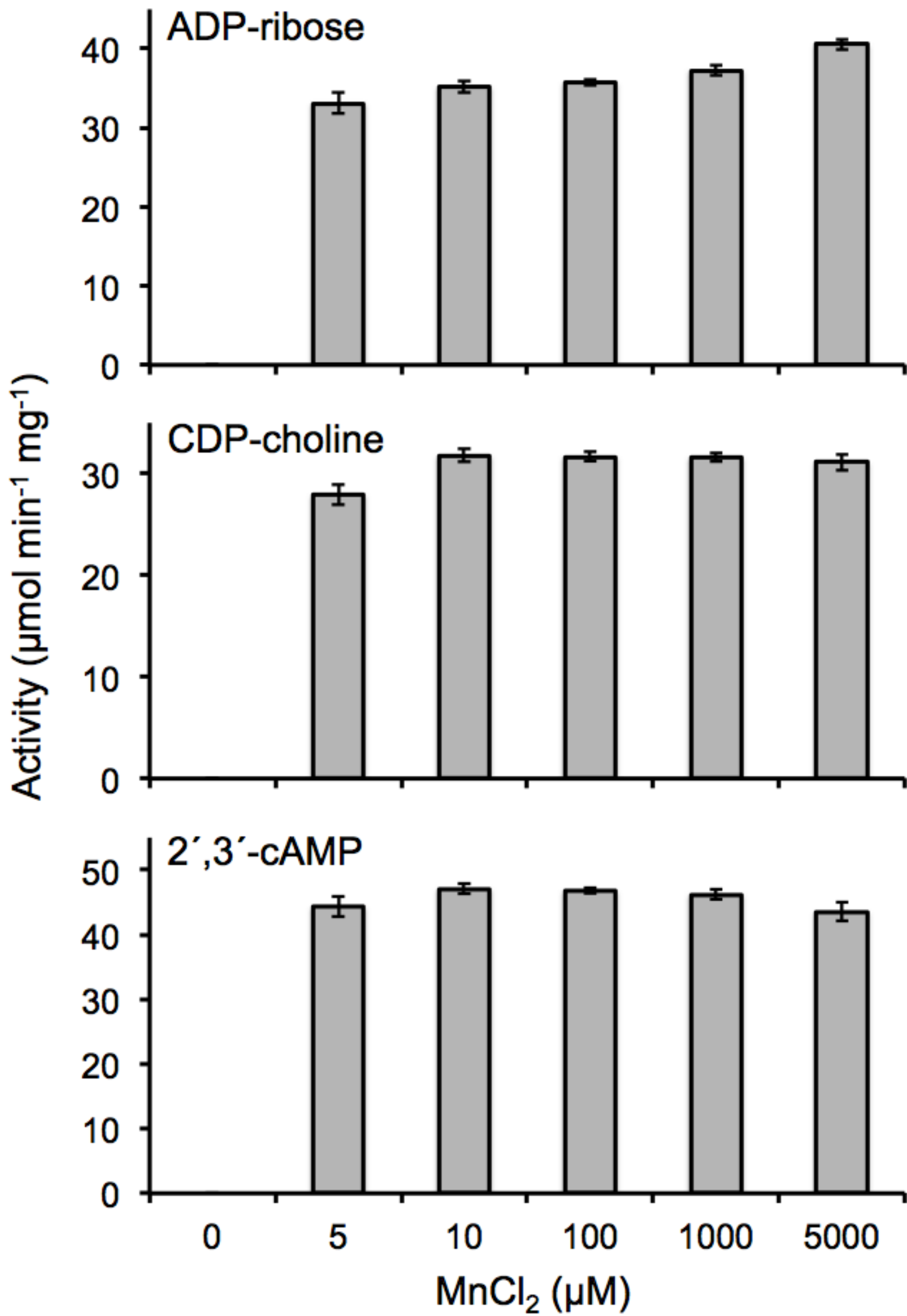

Supplement: S2 Fig — In this experiment the means ± S.D. were obtained in triplicate assays performed at pH 7.5, with 500 μM ADP-ribose or CDP-choline, or with 2500 μM 2´,3´-cAMP, in the presence of the indicated MnCl2 concentrations. The response of activity to Mn2+ concentration (5 μM-5000 μM) is representative of several other experiments performed under different experimental conditions. (PDF) [file pone.0118680.s006.pdf]

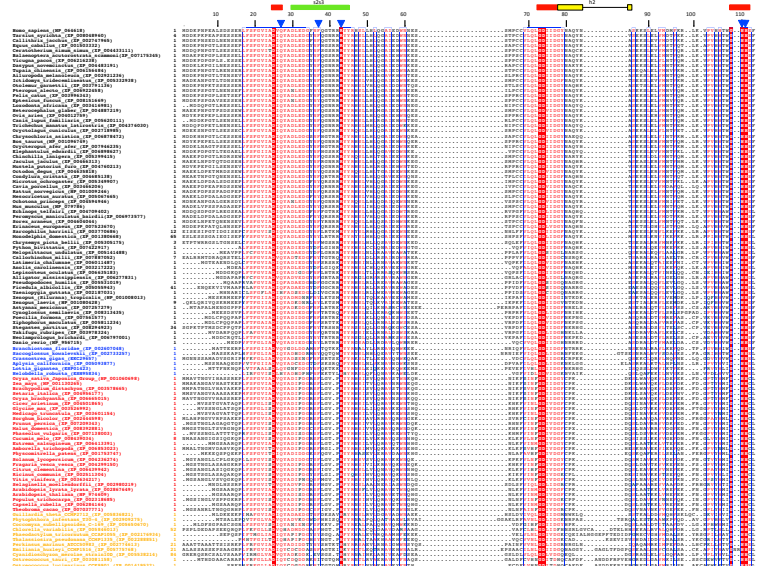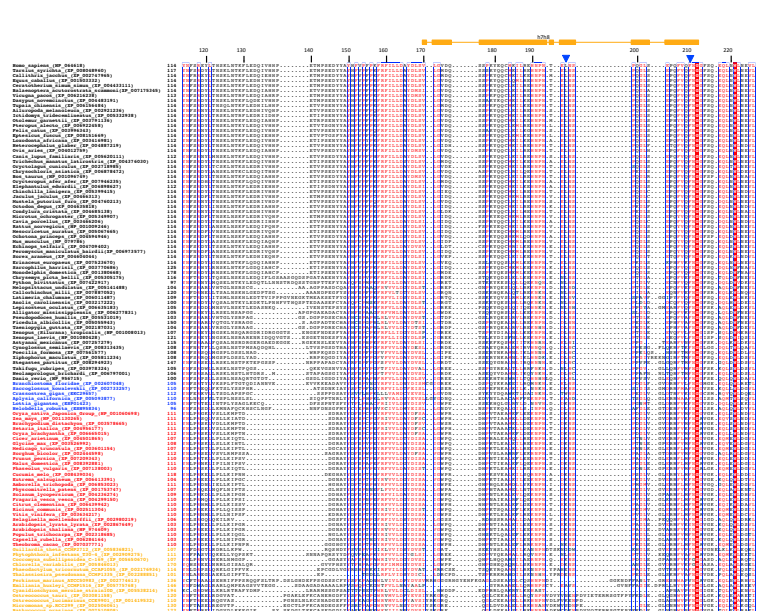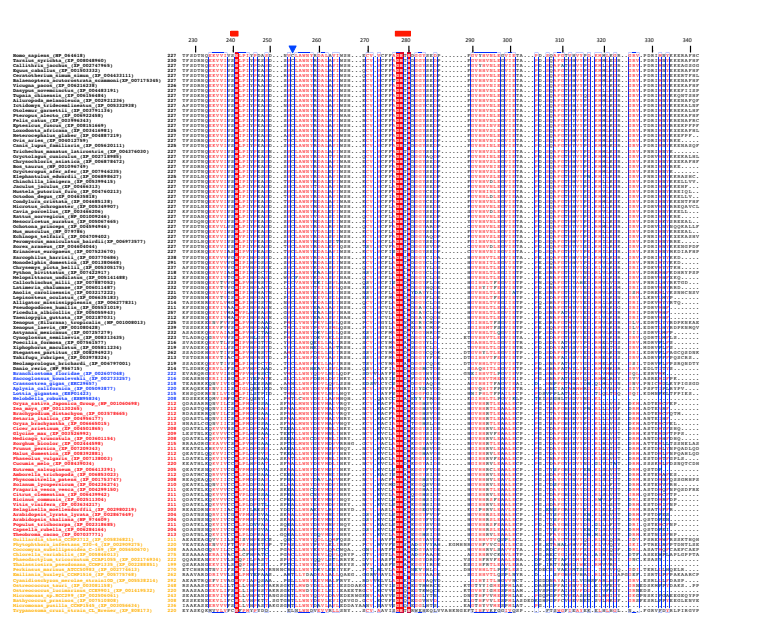

Supplement: S3 Fig — The names of species and GenBank accession numbers of the proteins are colored according to the following code: black, vertebrates; blue, invertebrate animals; red, plants; orange, protists. All the proteins were sorted out from a BlastP search [57] run on July 7, 2014. The proteins shown were selected applying criteria described under Materials and Methods, except for the selection of a few additional proteins: (i) the one from Mus musculus, despite being 96% identical to that from Rattus norvegicus, and due to its classification as the product of an immune gene [2, 37]; (ii) those from Bos taurus and Ovis aries, despite being 97% identical, because these species are included in NCBI RefSeq Genomes; (iii) those from the bivalve Crassostrea gigas, the snail Lottia gigantea, and the worm Helobdella robusta, despite not being yet recorded in NCBI_RefSeq, due to their importance as possible representatives of the protein family in invertebrate animal phyla. Residues identically conserved in all sequences are denoted by white letters on red background. Other regions of high sequence conservation are indicated by boxed red letters on white background. The numbers above the sequences correspond to the human protein. Blue triangles mark the amino acids that were mutated in this study. Red rectangles mark the five short regions that together form the disperse amino acid motif of the MDP superfamily. The unique regions s2s3, h2 and h7h8, typical of the ADPRibase-Mn family [52], are also indicated above the human protein. (PDF) [file pone.0118680.s007.pdf]

## ADP-ribose

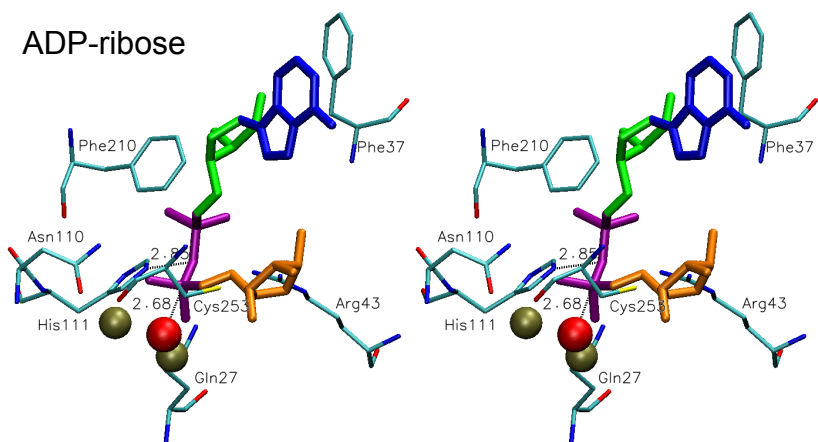

## cADPR

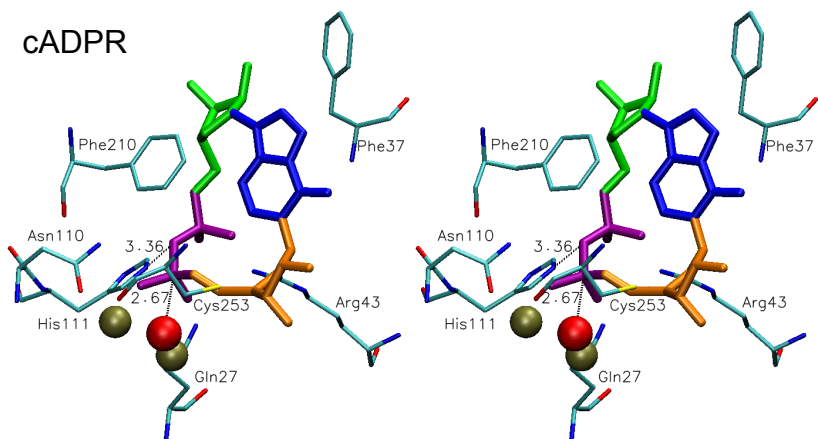

## CDP-choline

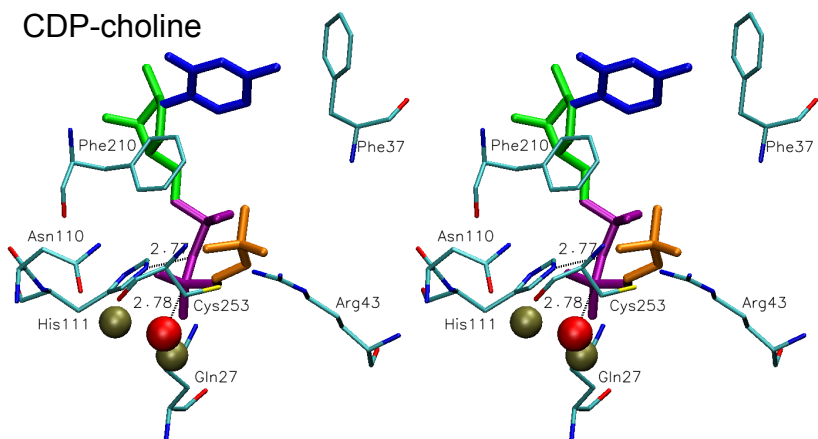

## 2',3'-cAMP

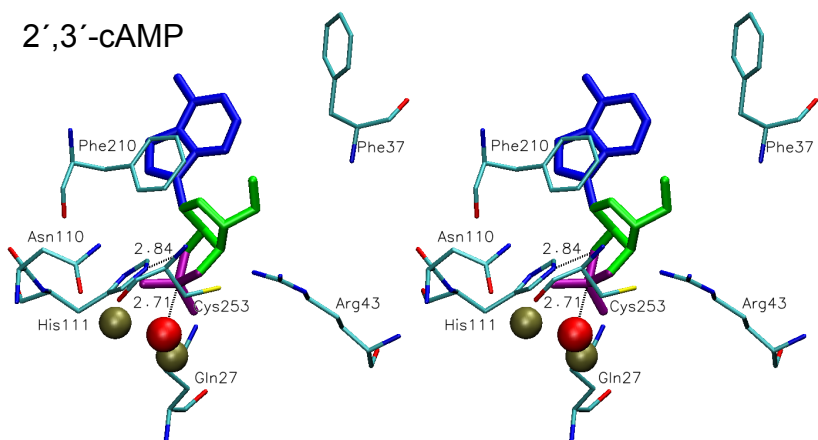

Supplement: S4 Fig — The views show metals, metal-bridging water, and amino acids identified by their interaction with docked ADP-ribose and tested by mutagenesis (see the main text for details). The models illustrate the hypothesis that the metal bridging water, according to its location, could be the attacking nucleophile in the ADPRibase-Mn reactions. The nucleophilic attack distances are drawn only to emphasize this hypothesis. (PDF) [file pone.0118680.s008.pdf]

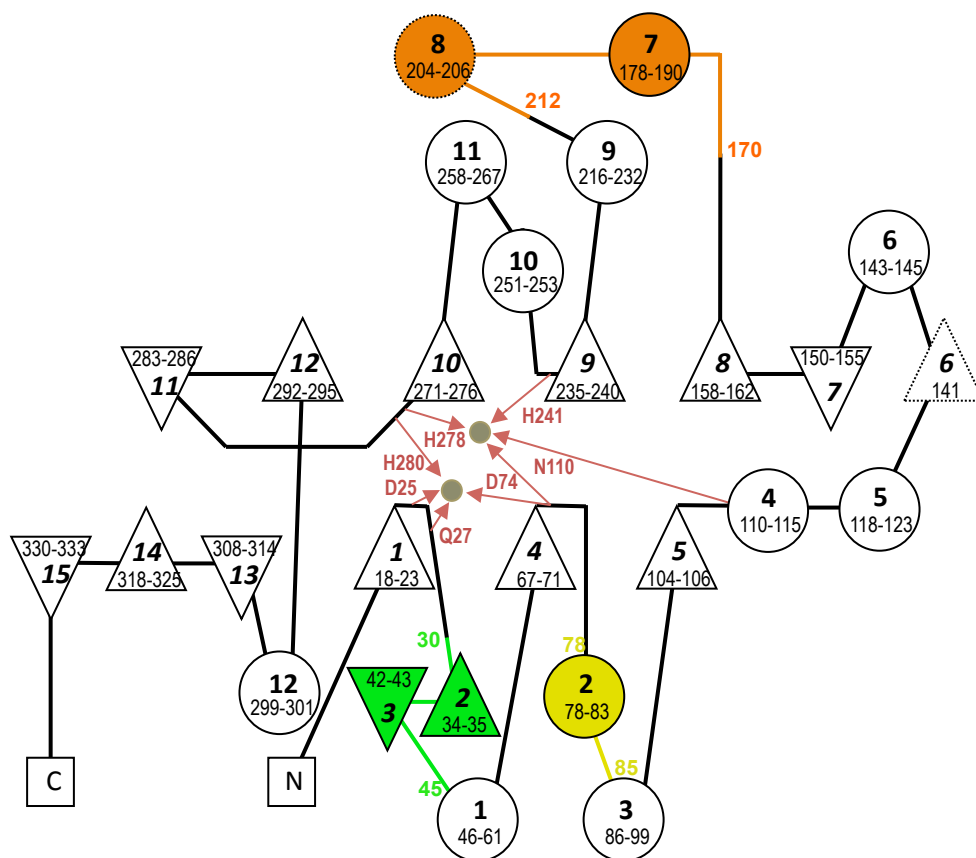

Supplement: S5 Fig — Structural elements (triangle, strand; circles, helix) are shown according to their numbering (strands 1–15 and helices 1–12) in zebrafish ADPRibase-Mn [52]. All the other numbers identify residues of human ADPRibase-Mn. The small golden circles are the metals of the dinuclear center; the eight amino acids of human ADPRibase-Mn coordinated to the metals are indicated. Strand 6 and helix 8 are present in the zebrafish protein; however, in the human protein, strand 6 is just a beta bridge and helix 8 was missing in the model downloaded from SWISS-MODEL to prepare the complete model structure of human ADPRibase-Mn (see Materials and Methods and Fig. 1), but appears in more recent models in the same repository [53]. (PDF) [file pone.0118680.s009.pdf]
