# Supplementary material for: Molecular Bases of Catalysis and ADP-Ribose Preference of Human Mn2+-Dependent ADP-Ribose/CDP-Alcohol Diphosphatase and Conversion by Mutagenesis to a Preferential Cyclic ADP-Ribose Phosphohydrolase
Source: PLoS One. 2015 Feb 18;10(2):e0118680. doi: 10.1371/journal.pone.0118680 (PMC4334965; doi:10.1371/journal.pone.0118680)
Supplement: S1 Table — The reverse primers were the reverse complements of those shown. (PDF) [file pone.0118680.s010.pdf]

Table S1 Forward primers used for site-directed mutagenesis.

Mutation	Forward primer
Q27H	CGTCATCGCAGATGTTCACTTTGCAGACTTAGAAGATGGC
F37A	GCAGACTTAGAAGATGGCTTTAATGCACAAGGAACCAGGCGG
F37Y	GCAGATCTAGAAGATGGCTTTAATTACCAAGGAACCAGGCGG
R43A	GGCTTTAATTTCCAAGGAACCAGGCGGGCATACTACAGACATAG
N110A	CATCATACATGGGGAGCTCATGAATTCTATAACTTCAGTAGAGAG
H111A	CATCATACATGGGGAAACGCTGAGTTCTATAACTTCAGTAGAGAG
H111N	CATCATACATGGGGAAACAATGAGTTCTATAACTTCAGTAGAGAG
L196A	CCAAATACGGAAGCGAATTCTCCTCAAGGACTTTCTGAGCCCCAG
L196F	GGAGCACAATCCAAATACGGAATTCAATAGCCCTCAAGGACTTTC
F210A	CAGTTTGTACAGGCTAATGGAGGATTCAGTCAAGAACAGCTAAAC
C253A	CCCGGACGCCTCTGACAATGTTGCCCTGGCCTGGAACTACAG

The reverse primers were the reverse complements of those shown.
