# Supplementary material for: Molecular Bases of Catalysis and ADP-Ribose Preference of Human Mn2+-Dependent ADP-Ribose/CDP-Alcohol Diphosphatase and Conversion by Mutagenesis to a Preferential Cyclic ADP-Ribose Phosphohydrolase
Source: PLoS One. 2015 Feb 18;10(2):e0118680. doi: 10.1371/journal.pone.0118680 (PMC4334965; doi:10.1371/journal.pone.0118680)
Supplement: S2 Table — In each case, enzyme purity was estimated by Coomassie blue-stained SDS-PAGE followed by image analysis quantitation. (PDF) [file pone.0118680.s011.pdf]

Table S2 Purity of the recombinant protein preparations.

Mutant ADPRibase-Mn	Purity (%)	Purity ratio versus wild type
Wild type	69.6	1.00
Q27H	71.4	1.03
F37A	54.1	0.78
F37Y	66.6	0.96
R43A	51.5	0.74
N110A	65.0	0.93
H111A	53.7	0.77
H111N	54.6	0.78
L196A	57.0	0.82
F210A	78.9	1.13
C253A	66.5	0.96
F37A+L196A	71.3	1.03
F37A+L196F	73.4	1.06
F37A+L196F+C253A	72.3	1.04

In each case, enzyme purity was estimated by Coomassie blue-stained SDS-PAGE followed by image analysis quantitation.
