# Supplementary material for: Molecular Bases of Catalysis and ADP-Ribose Preference of Human Mn2+-Dependent ADP-Ribose/CDP-Alcohol Diphosphatase and Conversion by Mutagenesis to a Preferential Cyclic ADP-Ribose Phosphohydrolase
Source: PLoS One. 2015 Feb 18;10(2):e0118680. doi: 10.1371/journal.pone.0118680 (PMC4334965; doi:10.1371/journal.pone.0118680)
Supplement: S3 Table — The activities were assayed in triplicate with 500 μM substrate in the presence of 5 mM MnCl2. The results are mean values with S.D. The sensitivity limit of the assay is indicated when no activity was detected. (PDF) [file pone.0118680.s012.pdf]

Table S3 Substrate specificity of human ADPRibase-Mn.

Substrate	Specific activity $\mu mol\ min^{-1}\ mg^{-1}$
ADP-ribose	52 ± 5
CDP-choline	46 ± 1
CDP-ethanolamine	36 ± 2
CDP-glycerol	42 ± 2
2´,3´-cAMP	21 ± 1
ADP	2.6 ± 0.5
cADPR	2.1 ± 0.2
ADP-glucose	≤ 0.05
UDP-glucose	≤ 0.05
CDP	≤ 0.05
CMP	≤ 0.05
AMP	≤ 0.05
3´,5´-cAMP	≤ 0.05

The activities were assayed in triplicate with 500 µM substrate in the presence of 5 mM $MnCl_2$. The results are mean values with S.D. The sensitivity limit of the assay is indicated when no activity was detected.
